# Modelling the effects of normal faulting on alluvial river meandering

**DOI:** 10.1002/esp.5315

**Published:** 2022-02-05

**Authors:** Hessel A. G. Woolderink, Steven A. H. Weisscher, Maarten G. Kleinhans, Cornelis Kasse, Ronald T. van Balen

**Affiliations:** ^1^ Faculty of Science, Earth and Climate Cluster Vrije Universiteit Amsterdam Amsterdam HV the Netherlands; ^2^ Department of Physical Geography, Faculty of Geosciences Utrecht University Utrecht TC the Netherlands; ^3^ TNO Geological Survey of the Netherlands Utrecht TA the Netherlands

**Keywords:** alluvial rivers, faulting, meandering, modelling, morphodynamics, morphology, neotectonic, tectonic

## Abstract

The meandering of alluvial rivers may be forced by normal faulting due to tectonically altered topographic gradients of the river valley and channel at and near the fault zone. Normal faulting can affect river meandering by either instantaneous (e.g. surface‐rupturing earthquakes) or gradual displacement. To enhance our understanding of river channel response to tectonic faulting at the fault zone scale we used the physics‐based, two‐dimensional morphodynamic model Nays2D to simulate the responses of a laboratory‐scale alluvial river with vegetated floodplain to various faulting and offset scenarios. The results of a model with normal fault downstepping in the downstream direction show that channel sinuosity and bend radius increase up to a maximum as a result of the faulting‐enhanced valley gradient. Hereafter, a chute cutoff reduces channel sinuosity to a new dynamic equilibrium value that is generally higher than the pre‐faulting sinuosity. A scenario where a normal fault downsteps in the upstream direction leads to reduced morphological change upstream of the fault due to a backwater effect induced by the faulting. The position within a meander bend at which faulting occurs has a profound influence on the evolution of sinuosity; fault locations that enhance flow velocities over the point bar during floods result in a faster sinuosity increase and subsequent chute cutoff than locations that enhance flow velocity directed towards the floodplain. This upward causation from the bend scale to the reach and floodplain scale arises from the complex interactions between meandering and floodplain and the nonlinearities of the sediment transport and chute cutoff processes. Our model results provide a guideline to include process‐based reasoning in the interpretation of geomorphological and sedimentological observations of fluvial response to faulting. The combination of these approaches leads to better predictions of possible effects of faulting on alluvial river meandering.

## Graphical abstract

Fault displacement leads to a transient response of the longitudinal bed profile and channel sinuosity. Channel sinuosity and the pacing of morphological responses are highly sensitive to the relative position within a meander bend at which faulting occurs. Upward causation from the bend scale to the reach and floodplain scale arises from the complex interactions between meandering and floodplain and the nonlinearities of the sediment transport and chute cutoff processes and has ramifications for reconstructions.

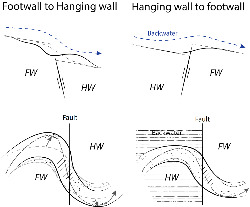

## INTRODUCTION

Meandering rivers form by interactions between flow, sediment, bed topology and channel curvature and migrate across their floodplains where they form a distinct morphology through meander initiation, expansion and cutoff (Hickin & Nanson, 1975; Hooke, 2007a,b; Weiss, 2016; Weisscher et al., 2019). Meandering rivers and their dynamics have been studied extensively, based on geomorphology, theoretical analyses, laboratory experiments and numerical modelling (Crosato, 2007, 2009; Friedkin, 1945; Hickin & Nanson, 1975; Hooke, 2007a,b; Howard & Knutson, 1984; Leopold & Wolman, 1957, 1960; Mosselman, 1995; Parker et al., 2011; Schumm, 1973; Sun et al., 1996; Sylvester et al., 2019; van Dijk et al., 2012).

**FIGURE 1:**
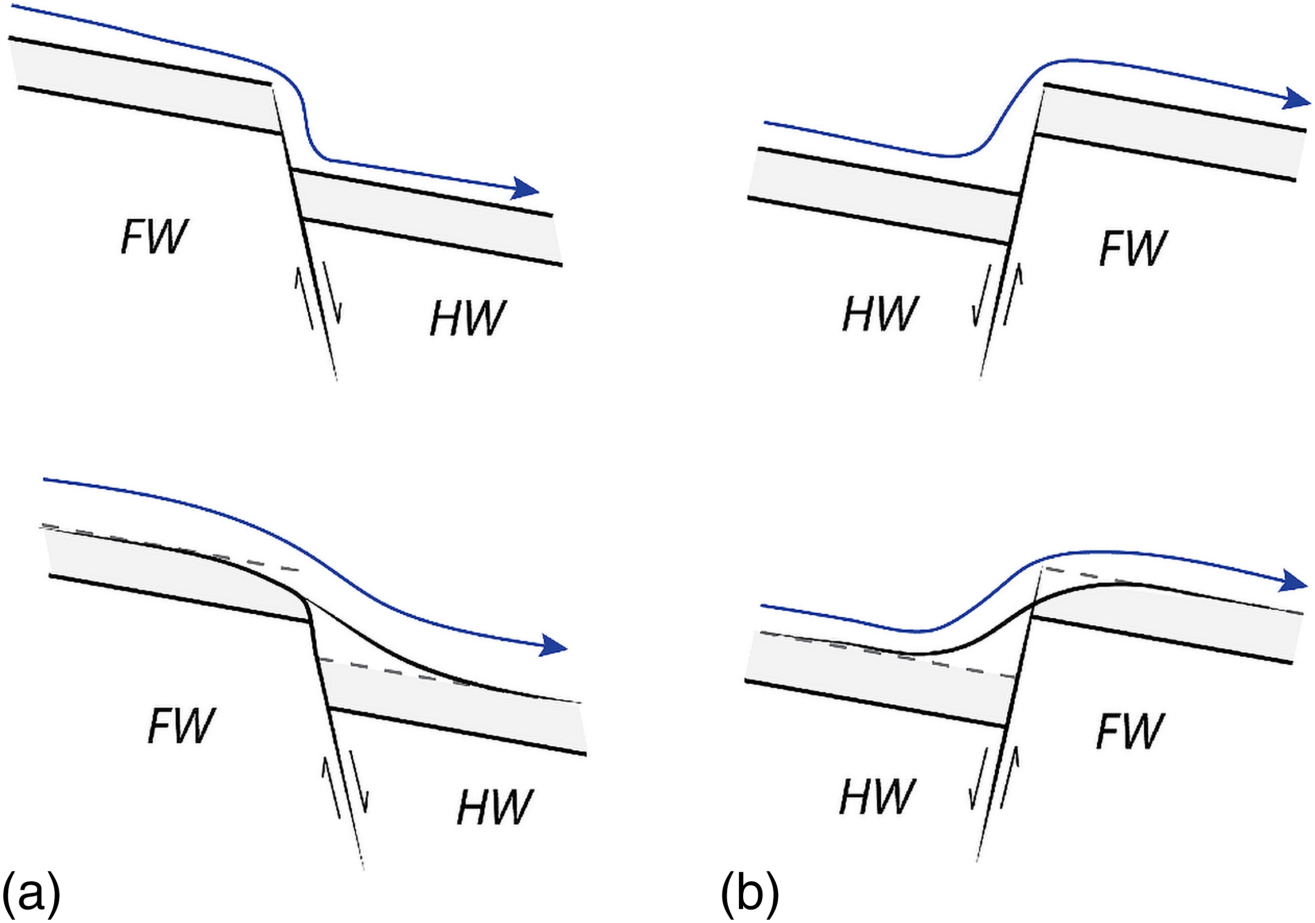
Potential effects of normal faulting on riverbed and flow deformation. (A) Normal fault downstepping in downstream direction (increases gradient). (B) Normal fault downstepping in upstream direction (reduces gradient). FW = footwall, HW = hanging wall, blue lines indicate water level. The lower two subfigures are later in time. Figure based on Holbrook and Schumm (1999) [Colour figure can be viewed at wileyonlinelibrary.com]

Many meandering rivers flow through tectonically active regions and it is critical to understand their responses to tectonic deformation to recognize and predict resulting changes in river dynamics and morphology. Faulting forces the morphodynamics of rivers by causing local changes to the topographic gradient of the river valley and channel (Holbrook & Schumm, 1999). Depending on the fault configuration, faulting can markedly impact the gradient of the riverbed and floodplain at the fault zone (Figure 1). Such changes in the topography of the river valley trigger a morphodynamic response of the river in order to restore the altered gradient. This morphodynamic response can be in the form of a longitudinal (bed) profile adjustment or in combination with a planform change of the river channel (Jorgensen, 1990; Schumm et al., 2002; Timár, 2003; Woolderink et al., 2021). A normal fault with the hanging wall in the downstream direction (i.e. downstepping in the downstream direction) enhances the fluvial gradient and hence degradation of the riverbed will occur upstream of the fault and aggradation downstream (Figure 1; Holbrook & Schumm, 1999). The opposite is expected for a fault that is downstepping in the upstream direction, which leads to aggradation of the riverbed in front of the fault and degradation downstream of a fault (Figure 1). Because of the transient nature (i.e. later erosion and sedimentation), the preservation of longitudinal riverbed deformations of alluvial rivers by faulting is rare in the sedimentary record (Holbrook & Schumm, 1999).

Channel adjustment patterns in response to faulting may range from a (local) shift in channel pattern (e.g. from scroll bar‐dominated to chute‐dominated meandering) to an intra‐pattern adjustment such as a change in sinuosity of a meandering river channel (Holbrook & Schumm, 1999; Ouchi, 1985; Timár, 2003). Moreover, faulting can alter the cross‐section (e.g. width‐to‐depth ratio) of a river via a change in gradient (Holbrook & Schumm, 1999). Such changes in channel width result in a changing bar pattern which might, in turn, alter floodplain morphology and channel migration (Kleinhans, 2010; Kleinhans et al., 2013). Hence, alluvial meandering rivers respond in various ways to faulting. However, each of these morphodynamic responses can also be the result of other causes (Holbrook & Schumm, 1999; Kleinhans, 2010; Ouchi, 1985), complicating derived relationships between faulting and river dynamics from the sedimentary and geomorphological records.

Normal faulting can affect river morphodynamics by either instantaneous (e.g. surface‐rupturing earthquakes) or gradual displacement. Both have a different effect on river morphodynamics, based on the ratio of vertical displacement and sediment transport capacity of the river. The reoccurrence interval of large surface‐rupturing earthquakes, that are able to produce co‐seismic vertical displacements of the earth's surface, is often relatively large. This hampers the possibility of collecting observational data of river response to co‐seismic vertical displacements. Slow tectonic deformation such as creep forces river morphodynamics on a timescale of thousands of years, obstructing the collection of observational data as well.

Over the last decades numerical modelling has provided the opportunity to study the effects of both intrinsic and extrinsic forcing factors on meandering river and floodplain dynamics (Crosato, 2007, 2009; Howard, 1992; Howard & Knutson, 1984; Iwasaki et al., 2016; Lancaster & Bras, 2002; Mosselman, 1995; Parker et al., 2011; Seminara, 2006; Stølum, 1996; Sun et al., 1996). Numerical modelling allows the isolation of effects of faulting, and many numeric modelling studies of alluvial river response so far focused on the long‐term basin‐scale effects of tectonics on alluvial architecture (Alexander & Leeder, 1987; Allen, 1978; Bridge & Leeder, 1979; Bryant et al., 1995; Mackey & Bridge, 1995). In contrast, model studies on the effects of faulting on more local alluvial meandering river morphodynamics (e.g. on the scale of multiple meander bends) are currently lacking.

Hence, our understanding of the morphodynamic and morphological responses of meandering rivers to various faulting scenarios on the scale of multiple meander bends around a fault zone is still limited. Therefore, the objective of this study is to determine the effects of both instantaneous and gradual faulting scenarios on meandering rivers at the scale of multiple meander bends along a normal fault.

To achieve this objective, we subject a river and its floodplain in a physics‐based, two‐dimensional morphodynamic model Nays2D (Shimizu et al., 2013) to faulting and compare the results to control runs to isolate observable effects of faulting to meandering river morphodynamics and morphology. Our study focuses on the sinuosity and longitudinal response of a laboratory‐scale alluvial river to various faulting and offset scenarios. This ‘landscape experiment’ is a simulation of an alluvial landscape that applies relaxed scaling (Kleinhans et al., 2014) and produces comparable sediment mobility, morphodynamic processes and complexity as that of a natural system (Kleinhans et al., 2014; Weisscher et al., 2019). Moreover, the advantage of numerical modelling a laboratory‐scale experiment is that the results of the model can be compared to past flume experiments (e.g. Jin & Schumm, 1987; Ouchi, 1985) and future experiments. This provides the opportunity to also study the effects of faulting on meandering river stratigraphy as well as transient responses as time is very much condensed. The dynamic system of an alluvial landscape in this study is subjected to instantaneous faulting over varying fractions of water depth and to gradual faulting on a timescale over which the river shows significant morphological change.

## METHODS

### Model description

Nays2D solves both depth‐averaged, nonlinear shallow water equations and the equations for bed load transport and bed level change (Shimizu et al., 2013). Bank erosion is incorporated in the model by a slope collapse module that corrects the bed elevation in case of oversteepening of the bank slope (Iwasaki et al., 2016). Weisscher et al. (2019) added colonization and mortality of vegetation to Nays2D. In this submodel, vegetation colonizes dry cells and is removed from drowned cells and from cells in which the erosion depth exceeds the rooting depth (Table 1). The settlement of (riparian) vegetation is used as a bar–floodplain conversion agent (Weisscher et al., 2019).

**TABLE 1:** Model settings for the scenarios in this study (after Weisscher et al., 2019)

Parameter	Value	Unit
Discharge *Q* (low–high)	1–6.4	L/s
Time step hydrodynamics/bed level change	0.02	s
Time step vegetation settling	3000	s
Channel width	0.5	m
Channel depth	0.04	m
Channel length	60	m
Aspect ratio (width/depth)	12.5	–
Grain size	0.76 × 10^−3^	m
Valley slope	2 × 10^−3^	m m^−1^
Inflow migration period *T*	1.53 × 10^5^	s
Inflow migration amplitude	0.75	m
Drag coefficient vegetation *C*_*D*_	1	–
Vegetation stem thickness *D*	0.5 × 10^−3^	m
Rooting depth	0.03	m
Manning's *n*	0.02	s m^1/6^
Shields number	0.07	–
Froude number	0.4	–

In this study, a model setup after Weisscher et al. (2019), who modelled a dynamic meandering river on a laboratory scale, was used. The focus of our study is on meandering rivers with downstream migrating meander bends.

Although the modelling of a laboratory‐scale alluvial river has the disadvantage that timescales of morphodynamic change may not accurately represent those of a real‐world meandering river, the key strengths of such a setup are that it can accurately model the river pattern and dynamics of a chute cutoff‐dominated meandering river with a self‐formed floodplain in terms of morphodynamic processes, complexity and sediment mobility as that of a natural system (Kleinhans et al., 2014; Weisscher et al., 2019). As the primary focus of our study is on modelling the general behaviour and morphodynamic responses of a meandering river to faulting in terms of sediment mobility and planform change, we choose to adopt the well‐established and validated model of a laboratory‐scale alluvial river which is based on real‐world river and experiment parameters instead of studying a specific (single) real‐world river.

**FIGURE 2:**
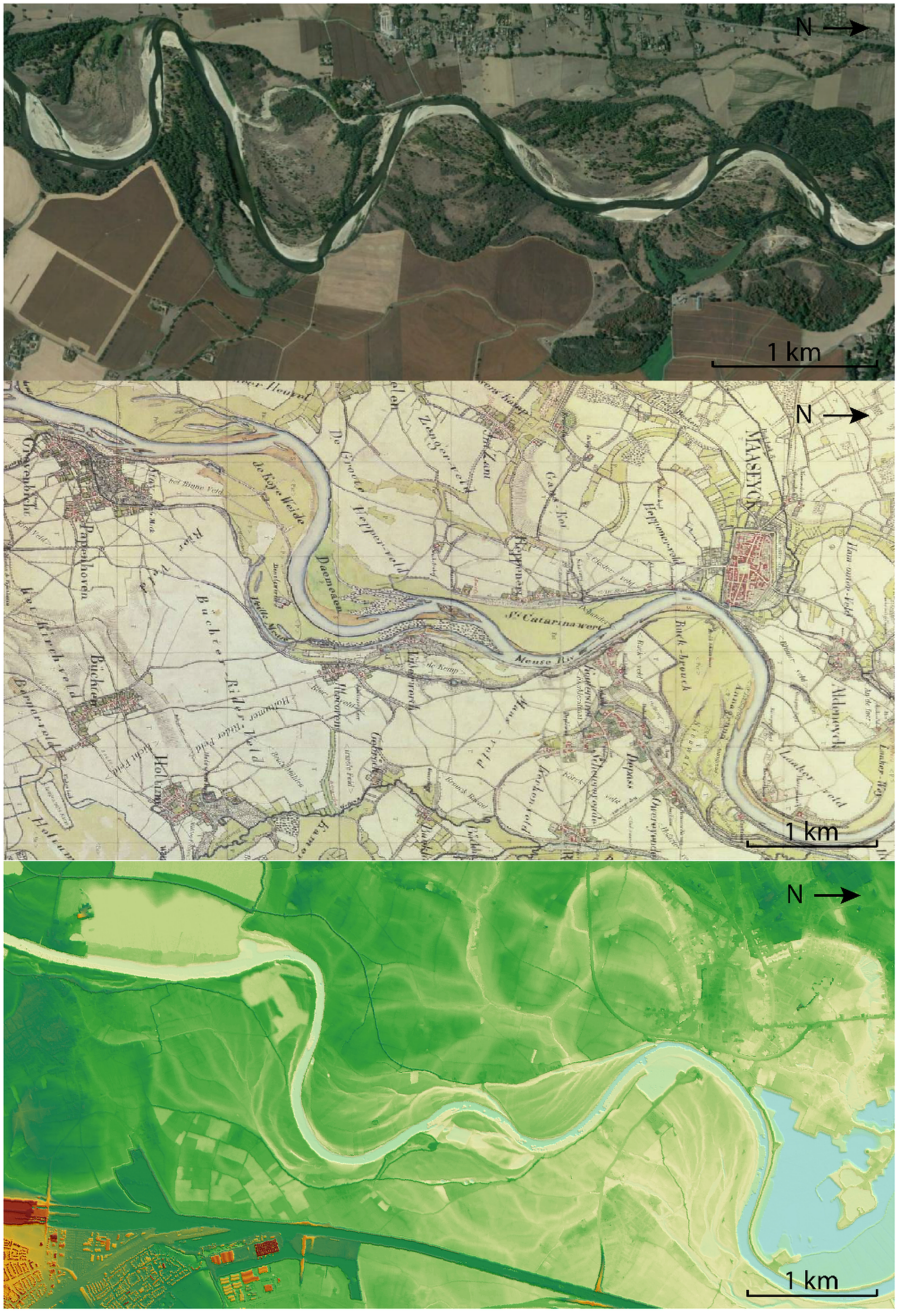
Top panel: Meandering reach with chutes and scrolls of the Allier River (France), upstream of Moulins. Middle panel: Historic map (Tranchot 1803–1820) showing the upstream part of the Meuse River in the southern Netherlands, dominated by scrolls and chutes. Bottom panel: Digital elevation model of the same stretch of the Meuse River (red is maximum elevation of ~41 m, blue is minimum elevation of ~20 m above Dutch ordnance level). Flow is from left to right [Colour figure can be viewed at wileyonlinelibrary.com]

The adopted model is based on the Allier River (France), the Otofuke River (Japan), the upstream part of the Meuse River in the Netherlands (Figure 2) and on past landscape laboratory experiments (van Dijk et al., 2012). All these rivers and the experiments are examples of gravel‐bed rivers, dominated by chute cutoffs and with similar morphological characteristics.

The model consists of a fixed rectilinear computational grid with 0.1 m square grid cells, representing a domain of 4.5 m wide and 60 m long. The initial straight channel was 0.5 m wide and 0.04 m deep. An initial lateral offset of +0.4 m was used for the 0.5 m fixed‐width inlet to steer morphological change. The slopes of the banks were 45°. The model setup applies bedload transport only (Meyer‐Peter & Müller, 1948), which is appropriate for low‐mobility gravel rivers. A sustained periodic inflow perturbation (e.g. movement of the inlet) was applied as a fraction of the width over a period in which significant meander migration occurred to ensure the initiation and persistence of dynamic meandering. This periodic perturbation was found to be a necessary condition for dynamic meandering in theory corroborated by numerical models and experiments, without acting as a forcing on the direction and rate of meandering (Lanzoni & Seminara, 2006; van Dijk et al., 2012; Weiss, 2016; Weisscher et al., 2019). Vegetation colonization is applied every 50 min, after which significant morphological change had happened, similar to changes during larger floods in natural rivers.

For our study, a normal fault was added to Nays2D. Grid cells downstream of the fault were displaced vertically to mimic fault movement as a fraction of the channel depth. In this way both the upstream and downstream boundary conditions remained unaltered. The amount of vertical displacement and the rate of deformation were varied to enable modelling of both instant and gradual faulting. To reduce computing time, we used the topographic output of an original model run after 167 h as the initial meandering planform for our faulting scenarios. At this time the (lateral) morphodynamic change of the meandering channel reached a dynamic equilibrium.

For all model scenarios we applied a minimum of 100 hydrological cycles of a 50 min hydrograph (83.3 h). The hydrograph varied between 1 and 6.4 L/s. The initial topography was run for 2.5 h to re‐establish hydrodynamics and vegetation cover on the loaded initial planform before a faulting perturbation was applied to the model.

### Data analysis

The bathymetry of the model scenarios was used for both qualitative and quantitative map comparison and was detrended with the initial valley gradient before the comparisons. The main channel is identified as the maximum value in each cross‐section of the detrended bed elevation times flow velocity to the power of three (Weisscher et al., 2019). This was empirically found to be the best indicator of channel position that excludes deep but abandoned channels and fast overbank flow (Weisscher et al., 2019). In order to exclude potential boundary effects, sinuosity of the main channel was determined in a zone extending from 10 m upstream to 10 m downstream of the fault. Sinuosity was computed by dividing the length of the main channel by the valley length. The initial and maximum meander amplitude and wavelength were measured using the main channel across the fault zone. Longitudinal profiles were determined along the main channel line. They show a smoothed profile of the detrended bed elevation.

### Model scenarios

Depending on the dip direction of the fault, normal faulting results in a relatively subsiding and uplifting riverbed in the part of the model downstream of the fault (Figure 1). In the description of the model scenarios, we therefore apply the terms ‘subsidence’ and ‘uplift’. A scenario without any faulting was conducted as a reference (L0). The subsidence scenarios represent a footwall to hanging wall transition (L3, L4, L7, L8, RC1–RC6; Table 2, Figure 1). The uplift scenarios model a hanging wall to footwall transition (L1, L2, L5, L6; Table 2, Figure 1). Both instantaneous and gradual faulting scenarios are simulated for the different fault configurations to study their effects on river morphodynamics (Table 2). Moreover, in both the subsidence and uplift scenarios the effects of a small (0.005 m or 1/8th channel depth of the model) and a large offset (0.015 m or 3/8th channel depth of the model) along the fault are studied (Table 2). In order to test the effects of the amount of vertical displacement we increased the fault throw for the subsidence scenarios incrementally by 0.005 m (or 1/8th channel depth of the model) to a maximum of 0.030 m (or 6/8th of the channel depth of the model; Table 2). Finally, the effect of the position of a fault along a meander bend is investigated for the subsidence scenarios to study the differential morphodynamic response (Table 2, F0–F2).

**TABLE 2:** Overview of deformation style, rate and fault location for the different scenarios in this study

Deformation style	Instant or gradual	Deformation rate (m/cycle or instant)	Total offset (m)	Total offset channel depth	Fault location (m)	Scenario
No deformation	–	–	–	–	–	L0
Subsidence	Gradual	−0.00005	−0.005	1/8th	32.0	L8
Subsidence	Abrupt	−0.005	−0.005	1/8th	32.0	L7/RC1
Subsidence	Gradual	−0.00015	−0.015	3/8th	32.0	L4
Subsidence	Abrupt	−0.015	−0.015	3/8th	32.0	L3/F0/RC3
Subsidence	Abrupt	−0.010	−0.010	2/8th	32.0	RC2
Subsidence	Abrupt	−0.020	−0.020	4/8th	32.0	RC4
Subsidence	Abrupt	−0.025	−0.025	5/8th	32.0	RC5
Subsidence	Abrupt	−0.030	−0.030	6/8th	32.0	RC6
Subsidence	Abrupt	−0.015	−0.015	3/8th	31.0	F1
Subsidence	Abrupt	−0.015	−0.015	3/8th	31.6	F2
Uplift	Gradual	0.00015	0.015	3/8th	32.0	L2
Uplift	Abrupt	0.015	0.015	3/8th	32.0	L1
Uplift	Gradual	0.00005	0.005	1/8th	32.0	L6
Uplift	Abrupt	0.005	0.005	1/8th	32.0	L5

The applied fault offsets are inspired on the reconstructed maximum vertical surface deformation along fault zones in the Lower Rhine Embayment (LRE) rift system (Camelbeeck et al., 2007; Houtgast et al., 2005; Kemna, 2005; van Balen et al., 2005, 2019; Westerhoff et al., 2008). Therefore, offsets of 0.4 and 1.2 m were used for the instant scenarios, which correspond to an approximate earthquake magnitude of ~6.5 to 6.8 (Wells & Coppersmith, 1994). The offsets correspond to approximately 1/8th and 3/8th of the channel depth of the Meuse River, respectively. Therefore, the same relative offsets of 1/8th and 3/8th of the channel depth of the initial model channel were used in the model simulations. The same total relative offsets were imposed for the gradual faulting scenarios, although these exceed the displacement rates of gradual faulting rates in the LRE, in order to compare the morphodynamic responses. The fault was positioned at the same location (32 m) in all scenarios, with the exemption of runs F1 and F2 (Table 2).

## RESULTS

**FIGURE 3:**
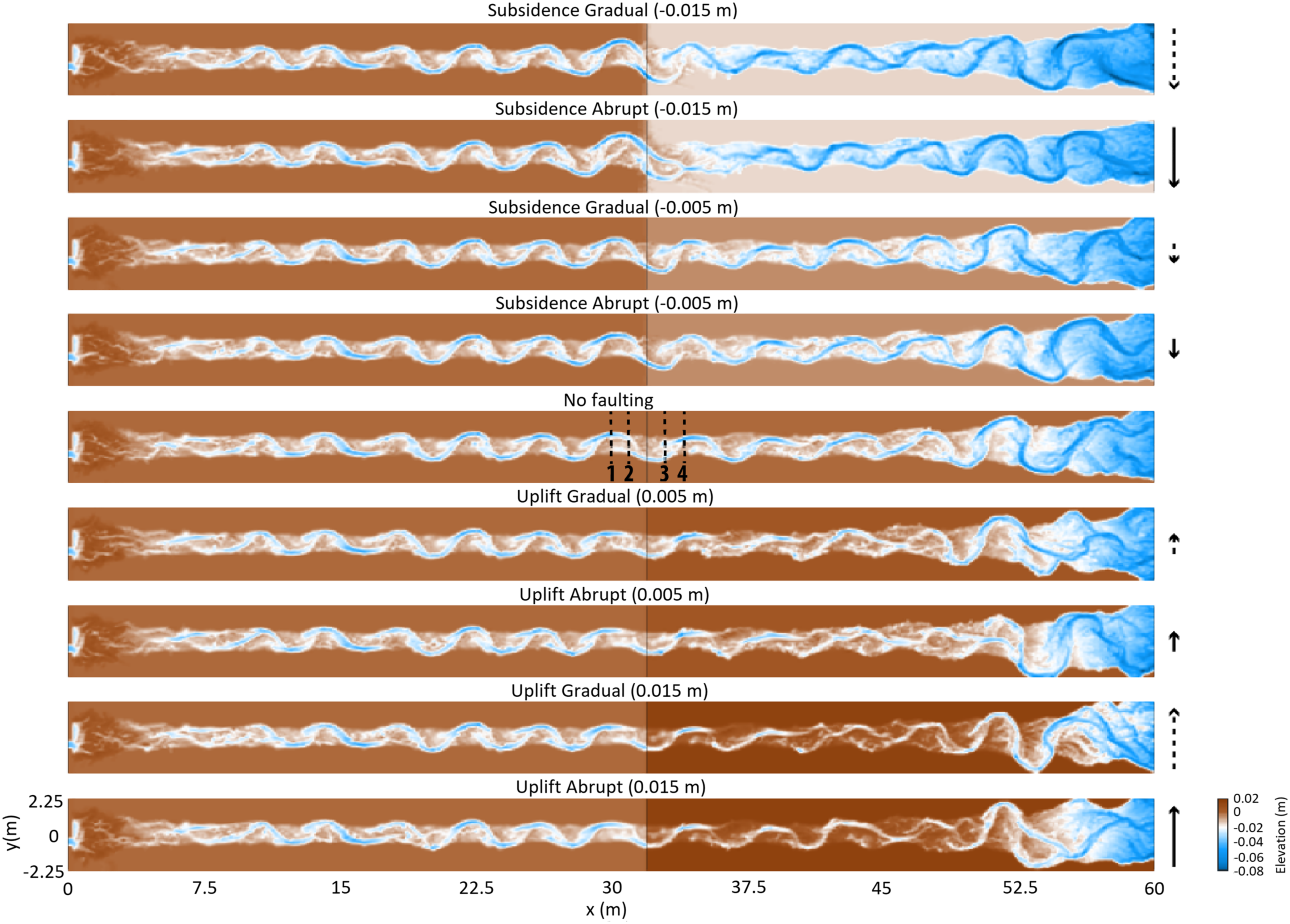
Morphology of the (detrended) riverbeds and floodplains for the model scenarios, at the end of the model run (*t* = 83.3 h). Arrows show the relative amount of uplift/subsidence for each scenario. Solid arrows are abrupt scenarios while dashed lines indicate gradual vertical displacement. Length of the arrows indicates the vertical displacement of either 1/8th or 3/8th of the channel depth. Black solid line represents the fault zone [Colour figure can be viewed at wileyonlinelibrary.com]

The results of the faulting scenarios are presented and subsequently interpreted and discussed below (Figure 3). First, the subsidence scenarios (normal faulting, downstream downstepping) are presented, followed by the scenarios that show the effects of increasing fault offset in the subsidence situation. Hereafter, the uplifting scenarios (normal faulting, upstream downstepping) are discussed. Finally, the effects of fault location within a meander on subsequent river morphodynamics are presented.

### Subsidence scenarios

#### Longitudinal profiles (normal faulting, downstream downstepping)

**FIGURE 4:**
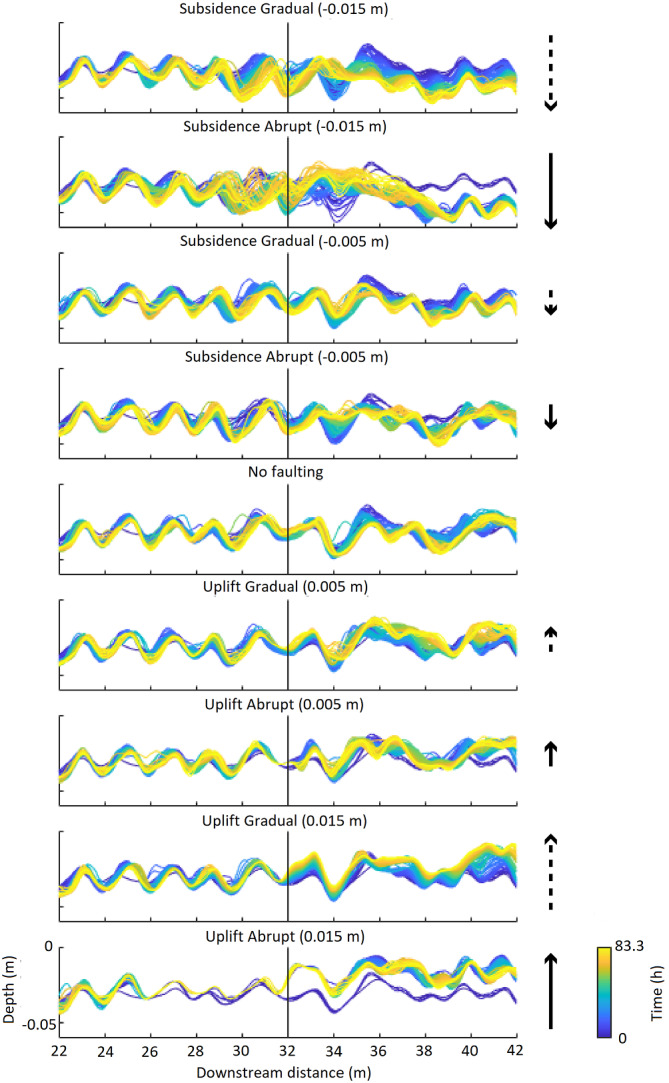
Longitudinal bed profiles over time of the faulting scenarios. Note that the subsidence scenarios show a clear adjustment of the riverbed to the faulting perturbation where erosion takes place upstream of the fault and sedimentation downstream of the fault. The uplift scenarios show a less clear adjustment to faulting. Arrows show the relative amount of uplift/subsidence for each scenario. Solid arrows are abrupt scenarios while dashed lines indicate gradual vertical displacement. Length of the arrows indicates the vertical displacement of either 1/8th or 3/8th of the channel depth. Black solid line represents the fault zone [Colour figure can be viewed at wileyonlinelibrary.com]

**FIGURE 5:**
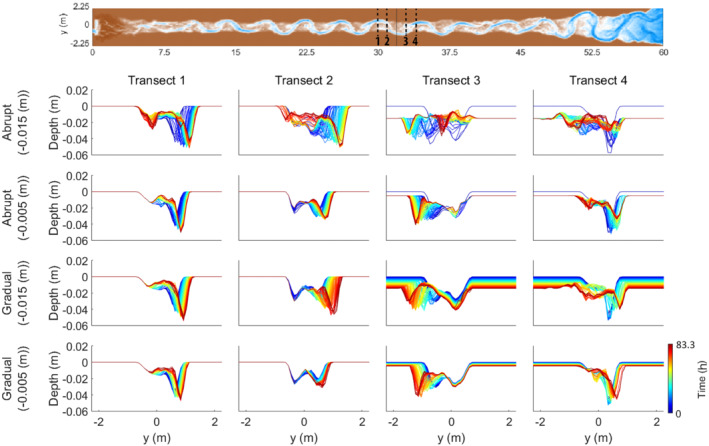
Cross‐sections over the floodplain at different distances to the fault for all of the subsidence scenarios. Panels show erosion upstream of the fault (transects 1 and 2) and sedimentation downstream (transects 3 and 4). Transects were placed at the nearest pool and riffle both upstream and downstream of the fault [Colour figure can be viewed at wileyonlinelibrary.com]

The subsidence scenarios show incision of the riverbed at and just upstream of the fault, which increases the channel depth relative to the floodplain (Table 2, Figure 4). The lowering of the bed level is larger near the fault zone compared to more upstream (Figure 4). The pools at the meander apexes show vertical erosion. The eroded sediment is deposited downstream of the fault, which increases the elevation of the channel bed and the channel width (Figures 4 and 5).

There is a difference in how the bed level changes between the abrupt and gradual displacement scenarios. Where the gradual subsidence scenarios show a slight lowering of the bed level at the riffle just downstream of the fault (i.e. at around 33 m), the abrupt scenarios show aggradation at this position. For the first outer bend downstream of the fault zone (i.e. at about 34 m), all subsidence scenarios show an increase in bed level and reduced channel depth over time compared to those immediately after the faulting perturbation (Figure 4). Aggradation of the bed level occurs to ~3 m (0.7 meander wavelength) downstream of the fault for the gradual subsidence scenarios. For the abrupt subsidence scenarios, this aggradation continues to ~6.5 m (1.5 meander wavelengths) downstream of the fault (Figure 4). Thus, the longitudinal bed profile responds to the faulting in the subsidence scenarios by incision upstream and aggradation downstream of the fault (Figure 4).

Incision of the riverbed upstream of the fault is the result of the locally increasing fluvial gradient and, hence, increased flow velocity and sediment transport capacity. Deposition downstream of the fault is the result of the change in gradient to the original gradient outside the fault zone. The extent of the adaption of the longitudinal profile is dependent on the rate of fault movement (Figure 4), which can be explained by the increase in gradient per unit time and the nonlinearity of sediment transport. The sudden increase in gradient, and thus flow velocity, leads to a larger amount of eroded sediment, and therefore also deposited volume, and hence downstream extent of channel bed aggradation, than in the continuous faulting scenarios.

#### Morphology and sinuosity

**FIGURE 6:**
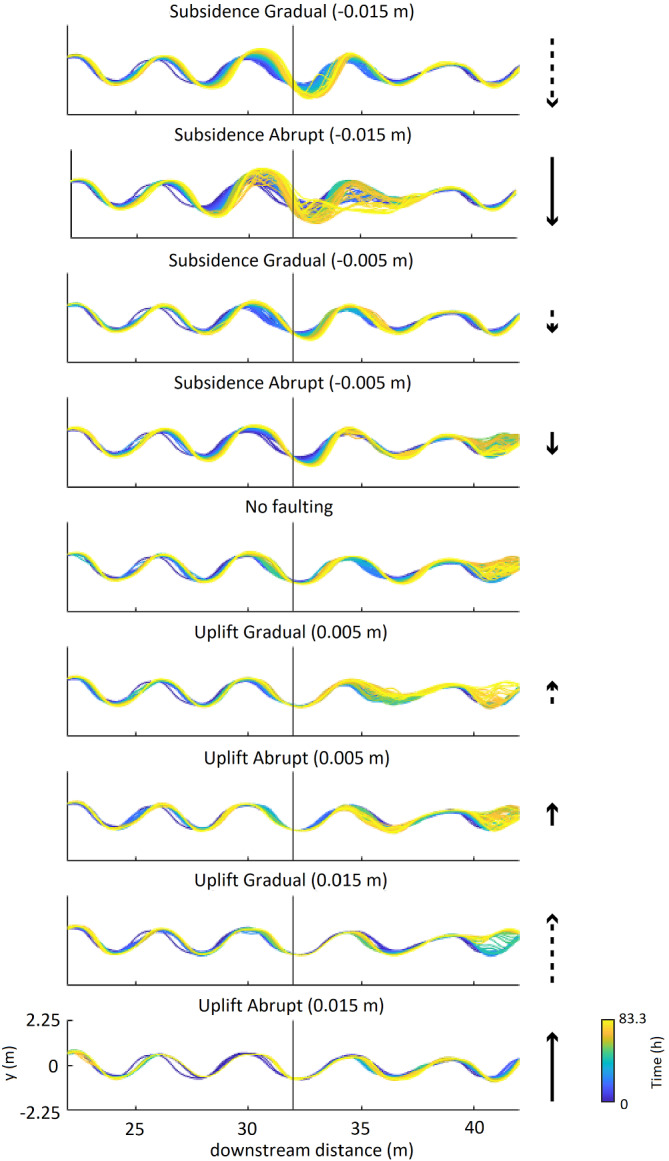
Position of the main channel over time for the model scenarios. Note that the change (or lack) of the main channel position occurs predominantly within 2 meander wavelengths around the fault [Colour figure can be viewed at wileyonlinelibrary.com]

The control scenario without any faulting (L0) shows an 11% increase in meander amplitude during the model run (Figures 3 and 6, Table 3). This increase in meander amplitude is most probably the result of intrinsic meander dynamics over time of the chute‐dominated meandering channel. The scenarios with relatively small subsidence (i.e. 0.005 m or 1/8th channel depth of the model) show that the meander amplitude increases ~20–30% at the fault zone due to lateral migration. The same is observed for the large gradual offset (0.015 m or 3/8th channel depth of the model), although here the amount of the meander amplitude change is higher (i.e. 52%; Figures 3 and 6, Table 3).

**TABLE 3:** Changes in meander amplitude for the model scenarios at the end of the run (83.3 h)

Scenario	Vertical displacement	Amplitude (m)	Change amplitude (m)	Change amplitude (%)	Normalized change amplitude
L0	No faulting	0.72	0.07	11.2	1.0
L08	Subsidence gradual (−0.005 m)	0.77	0.12	18.6	1.7
L07	Subsidence abrupt (−0.005 m)	0.85	0.21	32.4	2.9
L04	Subsidence gradual (−0.015 m)	0.98	0.34	52.0	4.6
L03	Subsidence abrupt (−0.015 m)	1.21	0.56	87.4	7.8
RC1	Subsidence abrupt (−0.005 m)	0.85	0.21	32.4	2.9
RC2	Subsidence abrupt (−0.01 m)	1.20	0.55	85.7	7.7
RC3	Subsidence abrupt (−0.015 m)	1.21	0.56	87.4	7.8
RC4	Subsidence abrupt (−0.02 m)	1.13	0.48	74.7	6.7
RC5	Subsidence abrupt (−0.025 m)	1.88	1.23	191.3	17.1
RC6	Subsidence abrupt (−0.03 m)	2.33	1.68	261.1	23.3
L02	Uplift gradual (0.015 m)	0.66	0.01	1.6	0.1
L01	Uplift abrupt (0.015 m)	0.60	−0.05	−7.8	−0.7
L06	Uplift gradual (0.005 m)	0.68	0.04	5.4	0.5
L05	Uplift abrupt (0.005 m)	0.66	0.02	2.3	0.2

**FIGURE 7:**
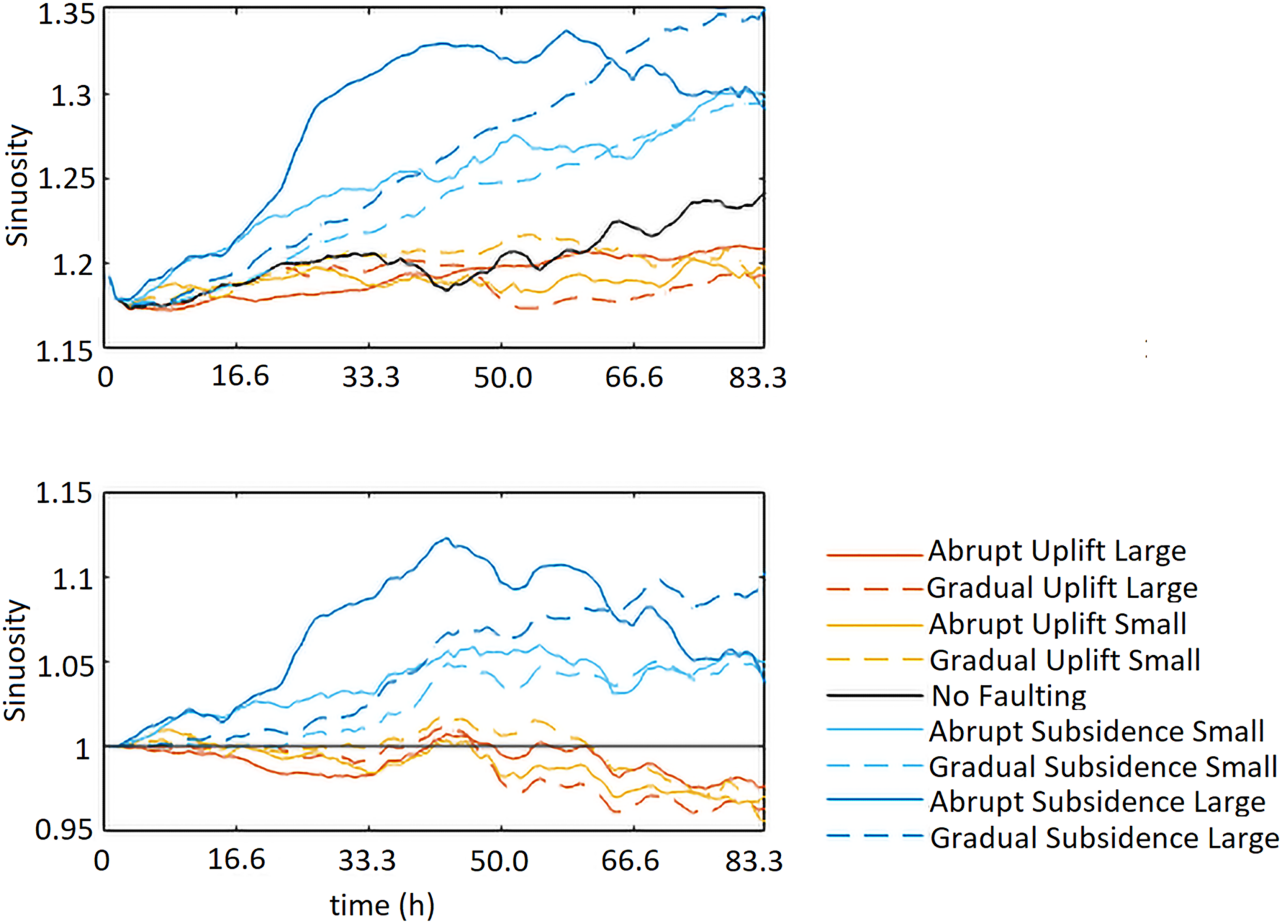
Sinuosity of the main channel over time for the model scenarios. Top panel shows the sinuosity profiles of model runs. Bottom panel shows the sinuosity profiles normalized to the reference run (L0) [Colour figure can be viewed at wileyonlinelibrary.com]

The results of the large instant offset simulations show a different pattern. In these results, the meander amplitude increases with a maximum of 87% of its original size, and the location shifts downstream till 62.5 h. Hereafter, a chute cutoff occurs and the main channel is straightened just downstream of the fault zone, which reduces the meander amplitude ~20% and doubles the wavelength of the meander (Figures 3 and 6, Table 3). The sinuosity at the fault zone increases from ~1.17 to 1.3 for all subsidence runs, except for the large gradual subsidence. In that simulation, sinuosity increases to 1.35 (Figure 7) at the location mainly centred around the fault zone (Figures 3 and 6).

**FIGURE 8:**
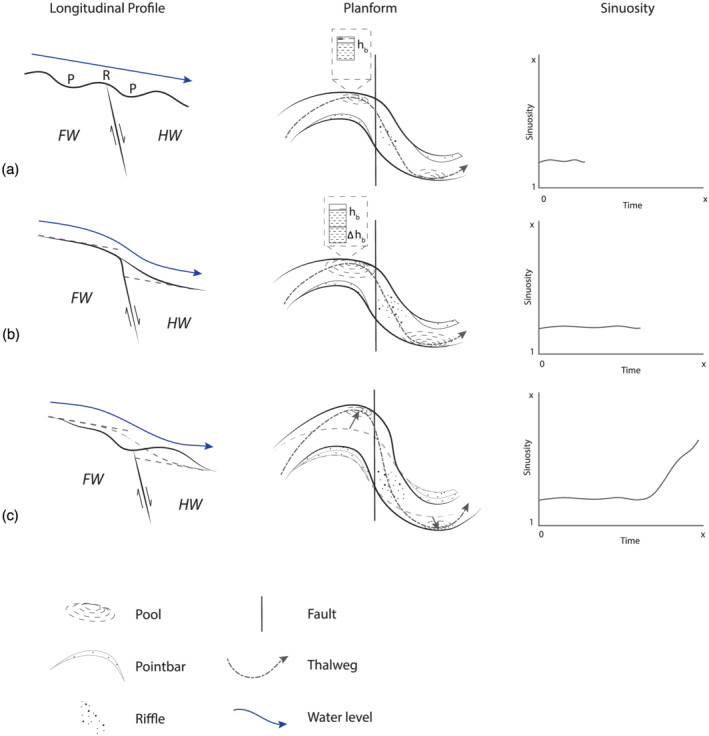
Schematic model of the effects of the subsidence faulting scenarios on the longitudinal riverbed profile and sinuosity increase. (A) Pre‐faulting scenario with riffle–pool morphology in the river channel. (B) Adaption of the longitudinal bed profile just after faulting with erosion of the bed upstream of the fault and sedimentation downstream. Scouring in the outer bend increases the height of the riverbank at these locations. (C) Further erosion and sedimentation in the longitudinal bed profile. The increase in bank height due to scouring in the outer bend leads to bank collapse and lateral migration of the river channel, which increases sinuosity of the channel. FW = footwall, HW = hanging wall [Colour figure can be viewed at wileyonlinelibrary.com]

Enhanced bank erosion occurs in response to the erosion and scouring at the pools in the outer part of the meander bends, which increases the outer bank slope and leads to mass failure when the angle of bed slope exceeds a critical angle (Figure 8B). The enhanced erosion of the outer banks leads to enhanced lateral bend migration and an increase of meander amplitude. This increases the sinuosity of the river channel. For the gradual subsidence scenarios, bank erosion and point bar accretion result in a more or less linear adaption of the sinuosity of the river channel (Figure 7). However, for the instant subsidence scenarios, sinuosity change shows a relatively fast increase in sinuosity after which a new (dynamic) equilibrium is reached (Figure 7). This difference in sinuosity response can be explained by the amount of gradient increase, which is much larger (100×) in the abrupt scenarios compared to the gradual scenarios.

Over time, erosion and sedimentation around the fault zone reduce the gradient over the fault. In turn, this reduces the lateral displacement rate and change in channel sinuosity (Figure 7). Moreover, by lengthening of the river channel by a sinuosity increase, the channel gradient is also reduced, complementing the in‐channel erosion and sedimentation. These processes can explain the limitation of further sinuosity increase of the river channel beyond the threshold of ~1.3 for the presented model runs.

As the sinuosity of the river channel increases due to outer bank erosion and lateral migration of the channel, sedimentation occurs in the channel (Figure 8). In addition, overbank flow occurs over the point bar during floods because of a gradient advantage compared to the main channel. As a result, a chute channel develops over the point bar, forming a bifurcation. The division of flow and sediment at this bifurcation results in sedimentation in the main channel, which is eventually closed, and a transformation of the chute channel to the new main channel occurs. This chute cutoff reduces the sinuosity of the river channel over the fault instantly (Figures 6 and 7).

#### Fault offset

**FIGURE 9:**
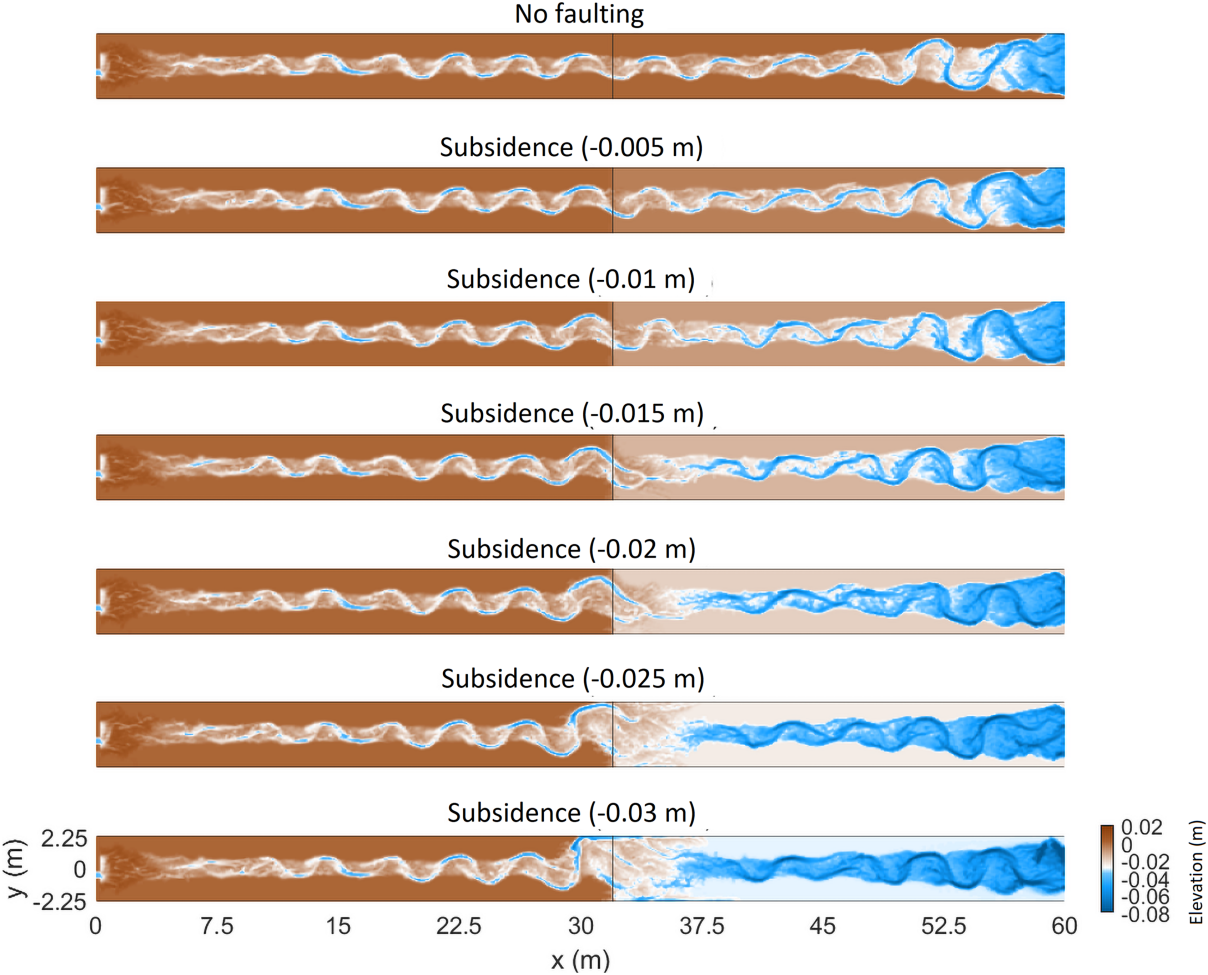
Morphology of the detrended bed elevation at the end of the model run for the scenarios that simulate incremental increase of subsidence. Morphodynamic change of the river channel increases with vertical offset [Colour figure can be viewed at wileyonlinelibrary.com]

**FIGURE 10:**
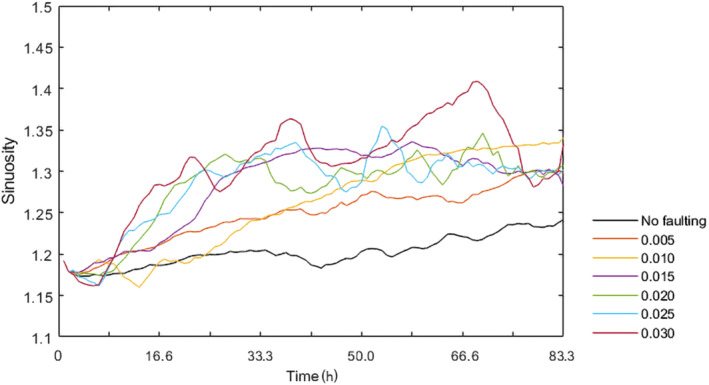
Sinuosity of the main channel over time for the incrementally increasing subsidence scenarios [Colour figure can be viewed at wileyonlinelibrary.com]

The abrupt subsidence scenario is used to test the effect of different amounts of fault offset as this scenario results in the most obvious sinuosity change (Figure 9). The amount of offset is incrementally increased 0.005 m (1/8th channel depth of the model) to a maximum offset of 0.03 m (6/8th channel depth). Figure 10 shows that the fault offsets of 0.005 and 0.010 m have a relatively constant sinuosity increase to ~50 and 66.7 h, respectively, before sinuosity stabilizes. The throw of the fault influences the rate of sinuosity increase, where larger offsets result in faster increase of sinuosity (Figure 10). The critical value of river sinuosity of our model runs lies, with the exception of the 0.030 m scenario, around 1.3 (Figure 10). Further increase of the channel sinuosity is associated with more variability of channel sinuosity. As discussed above, this leads to a chute cutoff and hence sinuosity decrease on a somewhat longer timescale.

The rate of sinuosity increase with increasing fault offsets can be explained by the fact that a larger offset results in a higher gradient and, therefore, a larger flow velocity. In turn, this leads to increased bank erosion, lateral displacement and thus a sinuosity increase of the channel.

**FIGURE 11:**
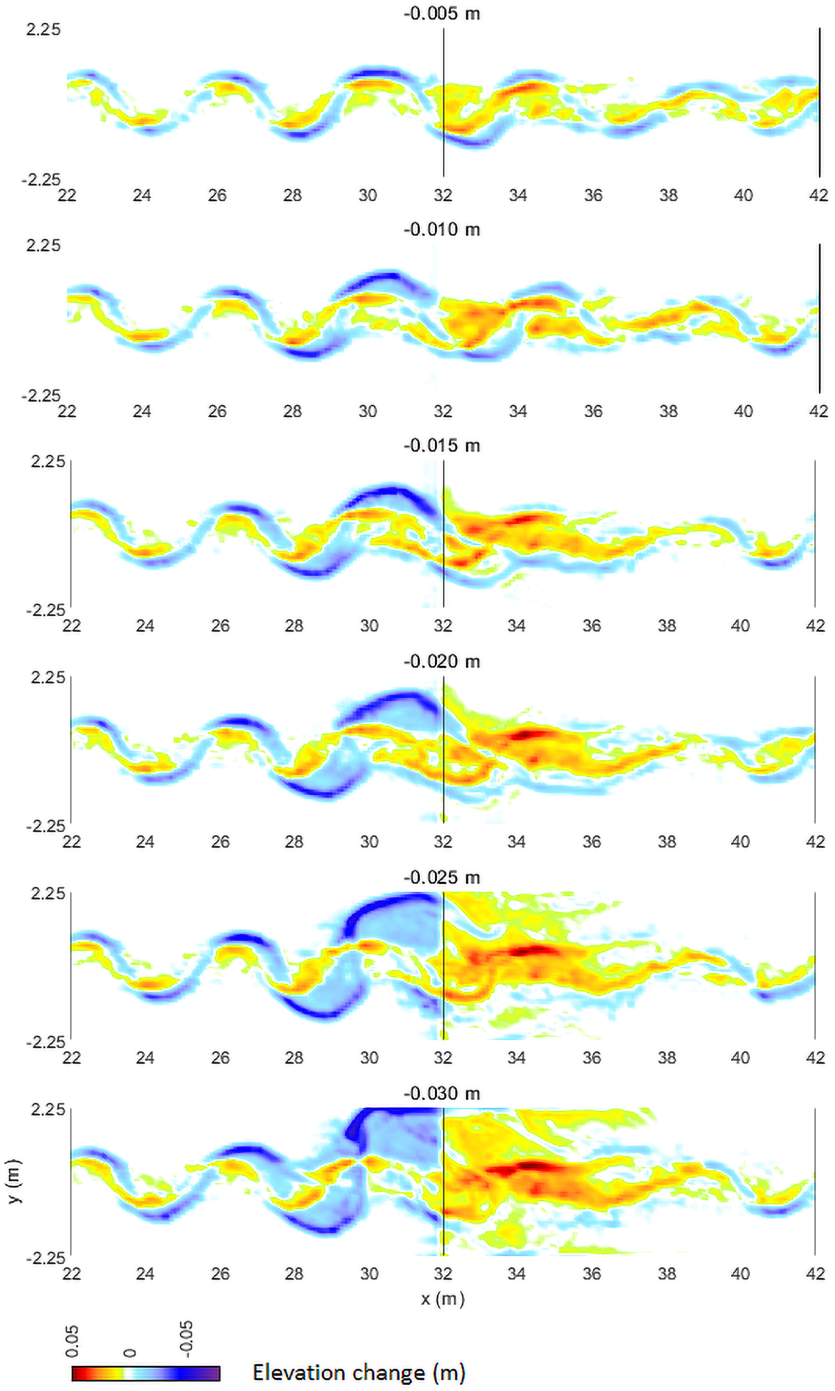
Change of morphology and bed elevation over the course of the model runs for the incrementally increasing subsidence scenarios (i.e. *t* = 83.3 – *T* = 0). Note that the largest subsidence scenarios result in a fluvial fan forming downstream of the fault that forces the channel to the side of the model domain, and thus affects the transverse channel location upstream of the fault [Colour figure can be viewed at wileyonlinelibrary.com]

The exception of the 0.030 m model run is probably the result of the large amount of sediment that is deposited downstream, at the foot of the fault (Figures 9 and 11). This sedimentation forms a fluvial fan, which forces the flow of the channel towards the side of the model domain. In an unconfined setting, this process might lead to a channel avulsion because the gradient towards the floodplain is significantly higher than of the fluvial fan.

### Uplift scenarios (normal faulting, downstepping in the upstream direction)

#### Longitudinal profiles

The effects of the uplift scenarios on the bed topography are inferred from longitudinal and perpendicular cross‐sections (Figures 4 and 13). Almost no change in bed level occurs just upstream of the fault on the hanging wall (Figure 4, Table 3). Moderate vertical erosion occurs in the outer meander bends on the footwall. On this relatively uplifted block the width of the channel does not change and, hence, the width‐to‐depth ratio decreases slightly (Figure 13). In general, the amount of vertical erosion increases further downstream of the fault location (i.e. within several metres of the fault; Figure 13).

**FIGURE 12:**
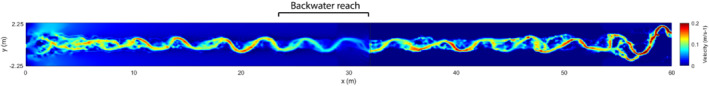
Example of the backwater that develops in front of the fault in the uplift scenarios. Velocities are reduced over a zone of ~2 meander wavelengths upstream of the fault [Colour figure can be viewed at wileyonlinelibrary.com]

The consistency in bed topography on the hanging wall can be attributed to the development of a backwater in front of the fault in these scenarios, which is caused by the obstruction to flow posed by the uplifted downstream footwall. The backwater effect reduces flow velocities in the channel on the hanging wall and, during floods, over the point bar and floodplain (Figure 12). The decrease in flow velocity reduces morphodynamics of the river channel in this reach. This process of decreasing flow velocity is most pronounced for the abrupt faulting scenarios, as this results in the most pronounced obstruction to flow in the river channel (Figure 12). The upstream length to which the backwater reaches depends on the amount of fault offset. For the relatively large instant faulting scenario (0.015 m or 3/8th model channel depth), this is ~8 m upstream of the fault, which is approximately 2 meander wavelengths (Figure 12). On the footwall, not affected by a backwater effect, flow velocities are not altered, resulting in moderate vertical erosion in the outer meander bends on this block.

**FIGURE 13:**
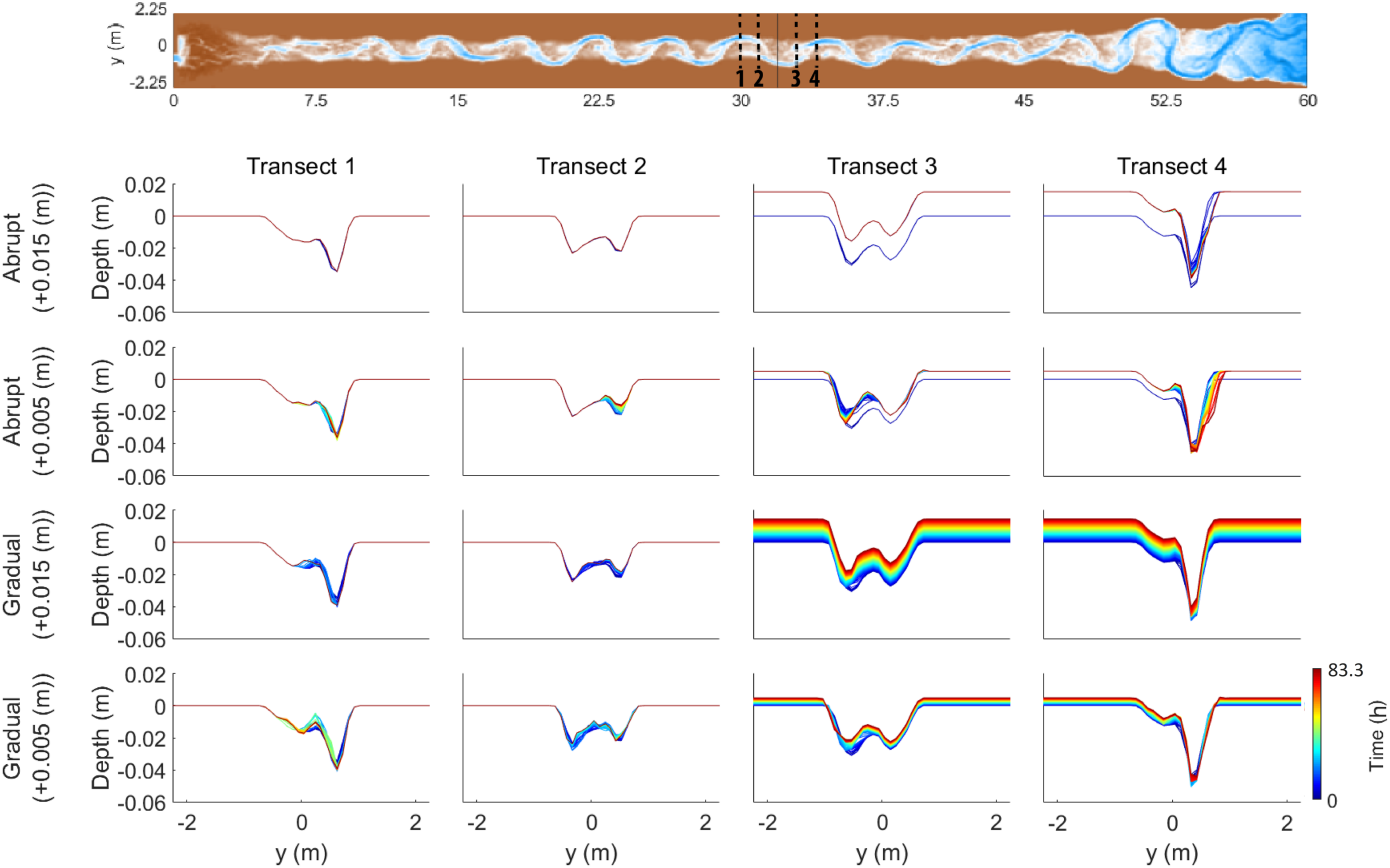
Cross‐sections over the floodplain at different distances to the fault for all of the uplift scenarios. Note the lack of change, especially for the abrupt scenarios, in front of the fault. Transects were placed at the nearest pool and riffle both upstream and downstream of the fault [Colour figure can be viewed at wileyonlinelibrary.com]

#### Morphology and sinuosity

The scenarios with relative uplift show that the main channel remains at a fixed position around the fault (Figure 6). An exception is the meander at ~27.5 m, which experiences a minor downstream displacement (Figure 6). Compared to the control run, the uplift scenarios show a smaller amplitude change (Table 3). As in the control run, the meander wavelength remains constant (Figure 6).

The sinuosity of the channel remains fairly constant over time (i.e. between ~1.17 and 1.20; Figure 7), especially for the first 33.3 h. After 41.6 h, the sinuosity of the reference run increases from 1.19 to 1.24. The uplift scenarios, however, show sinuosity values that remain more or less stable (Figure 7).

The lack of sinuosity increase in the uplift scenarios can partly be attributed to the backwater effect, which reduces flow velocity and hence decreases the amount of bank erosion and thus lateral migration rate of the channel. On the footwall, sediment concentrations are less than the transport capacity due to the reduced flow velocities in the backwater on the hanging wall. This leads to erosion in the channel and hence to slight channel straightening (Figure 6). The fault displacement makes the backwater reach on the relatively subsiding hanging wall more prone to flooding. In extreme cases even a lake develops, as in the abrupt large offset scenario (Figure 12).

### Fault location

**FIGURE 14:**
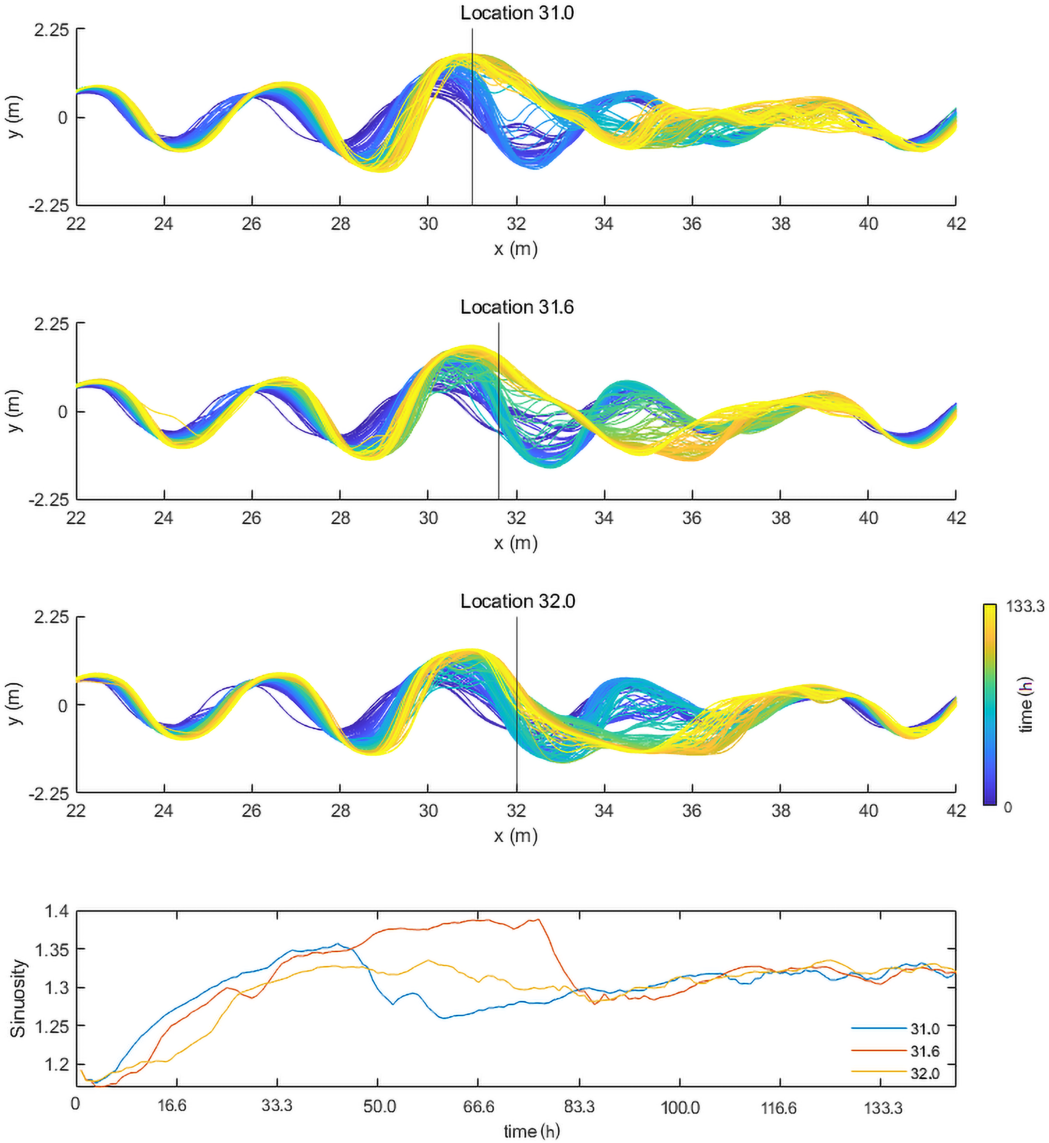
Evolution of the position and sinuosity of the main channel over time for the scenarios with different fault locations [Colour figure can be viewed at wileyonlinelibrary.com]

The effect of fault location was studied by using two additional scenarios (i.e. fault location at 31.0 and 31.6 m; Figure 14) with an instant fault offset of 0.015 m (3/8th channel depth of the model) using the subsidence setup. The first 41.7 h of both scenarios show a similar increase in sinuosity from ~1.18 to 1.33. However, hereafter the sinuosity changes of the different scenarios start to deviate (Figure 14). The scenario with a fault at 31.0 m shows a chute cutoff of the channel occurring at 45.8 h, which reduces the sinuosity to 1.27 within 8.33 h. Subsequently, sinuosity increases again to 1.31 at the end of the model run. The scenario with the fault at 31.6 m predicts sinuosity to increase for 58.3 h, and then the change in sinuosity decreases and sinuosity stabilizes around 1.39. However, at 76.7 h a chute cutoff reduces the sinuosity of the channel to 1.31. The standard fault location at 32.0 m shows a relatively stable sinuosity of 1.33 up to 60 h, after which it decreases to 1.29 to return to a sinuosity of ~1.33 at the end of the run. Overall, the different positions of the fault locations lead to a similar end result (i.e. a chute cutoff and sinuosity of ~1.33). However, substantial differences are observed for the time‐progressive evolution.

**FIGURE 15:**
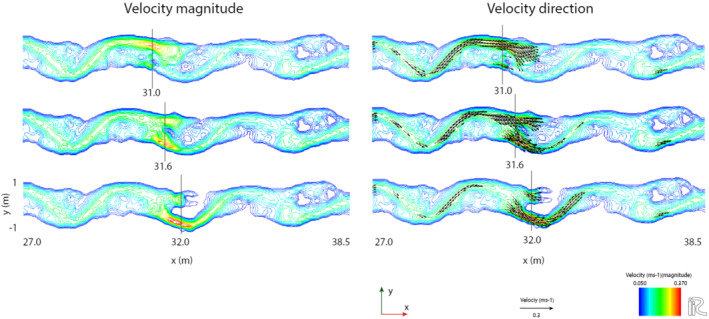
Velocity magnitude and direction of flow in the model during high discharge for the different fault location scenarios [Colour figure can be viewed at wileyonlinelibrary.com]

The cause for the observed differences in sinuosity response is best explained by the velocity maps of the flow after the fault displacement (Figure 15). The local increase in velocity at the fault location (left panels, Figure 15) is the result of an increased gradient. The location of 31.0 m positions the fault at the downstream end of the bend apex. Subsidence of the right‐hand side of the model domain therefore leads to a superposition of the upstream meander compared to the meander bend downstream of the fault (Figure 15). The combination of these factors makes the flow after fault displacement largely directed over the point bar downstream of the fault (right panels, Figure 15). This overbank flow, which has an increased velocity due to the increased gradient, leads to erosion of the point bar and chute channel development. Therefore, the sinuosity profile of the fault location at 31.0 m shows a relatively fast chute cutoff development (i.e. after 45.8 h; Figure 14, bottom panel).

The fault position at 31.6 m is located at the inflection point of the meandering channel. Although the same response as for the fault location at 31.0 m is partially observed for this fault position, a large part of the flow is directed into the main channel towards the next meander bend (Figure 15). This results in a longer period of bank erosion, lateral migration and hence meander curvature before the gradient advantage over the point bar becomes high enough for a chute cutoff to occur.

The fault location at 32.0 m positions the fault near the outer bend of the meander (Figure 15). In this case the fault offset results in a direction of the flow (with increased flow velocity) towards the outer bank of the meander. Here, scouring takes places which eventually leads to a sinuosity increase (Figure 14, bottom panel). However, the flow direction towards the outer bend is towards the vegetated, erosion‐resistant floodplain instead of the point bar and therefore does not contribute to the development of a chute channel. The fact that such a chute cutoff does eventually develop for the scenario with the fault location at 32.0 m can be attributed to the increased gradient advantage over the point bar which forms as a result of increased bend curvature and downstream bar formation.

## DISCUSSION

### Comparison to field and flume studies

**FIGURE 16:**
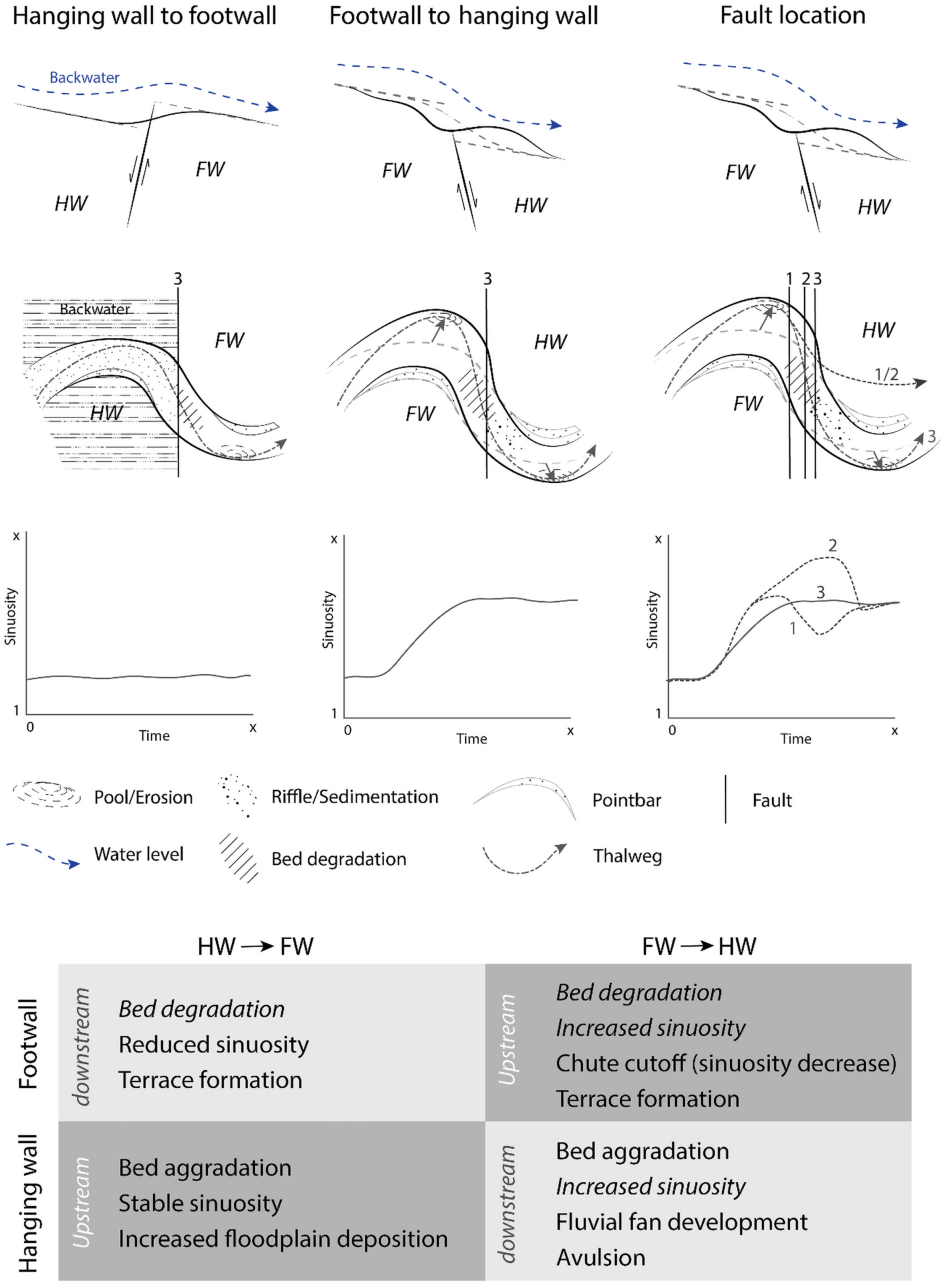
Overview of possible morphodynamic responses and resulting morphology of an alluvial river to various faulting scenarios. Italic type in the bottom panel indicates reduced preservation potential. Regular type indicates increased preservation potential. HW = hanging wall, FW = footwall [Colour figure can be viewed at wileyonlinelibrary.com]

The model results show that a changing valley gradient over a fault leads to both longitudinal erosion and aggradation of the riverbed (Figure 16). Erosion of the footwall and aggradation on the hanging wall of a fault are commonly observed responses to a gradient increase (i.e. a normal fault downstepping in the downstream direction) (Gasparini et al., 2016; Holbrook & Schumm, 1999; Parker, 2004). The shallowing and widening of the channel in our model results just downstream of the fault were also observed in both experimental and field studies for zones of relative subsidence and lowered slopes (Holbrook & Schumm, 1999; Jin & Schumm, 1987; Jorgensen, 1990; Ouchi, 1985).

An increase in sinuosity in response to a faulting‐induced steepening of the valley floor, as shown for the subsidence scenarios (Figures 3, 6 and 16), is observed frequently in the geomorphological record as well (Gomez & Marron, 1991; Ouchi, 1985; Petrovszki et al., 2012; Schumm et al., 2002; Timár, 2003). However, the model shows that a continued increase in sinuosity leads to a chute cutoff which reduces channel sinuosity (Figure 14). Therefore, the posed relationship between gradient and river channel sinuosity increase of a meandering river with downstream migrating meander bends is only valid up to a maximum threshold of bend sharpness (cf. Holbrook & Schumm, 1999; Schumm & Khan, 1972; Woolderink et al., 2021). Moreover, if faulting rates become too high relative to the sediment transport capacity of the river, excess sedimentation on the hanging wall leads to channel avulsion (Figure 11). These results are in agreement with Schumm et al. (2002), showing that the rate of deformation (i.e. faulting) influences the river response.

Flooding of the backwater reach was observed as a response to a normal fault downstepping in the upstream direction, as observed in field studies (Holbrook & Schumm, 1999). Such flooding or lake development often results in increased accumulation of (fine) sediments (Figure 16), due to the reduced flow and sediment transport capacity, at the riverbed and floodplain in the backwater reach (Holbrook & Schumm, 1999).

Increased incision and decreased width‐to‐depth ratios, such as in the predictions for the uplift scenarios, are frequently observed responses of river channels that transverse zones of uplift (Holbrook & Schumm, 1999; Jorgensen, 1990; Woolderink et al., 2018). A decreased channel sinuosity over a zone of relative uplift was, for example, observed in the geomorphological record of the Meuse River in the LRE rift system (Woolderink et al., 2018). However, anomalies in channel width, depth and width‐to‐depth ratio alone are not sufficient indicators for tectonic perturbation in field studies as these characteristics are also dependent on variables such as bankfull discharge, sediment load and type, vegetation patterns, floodplain and bank material and confluences or bifurcations (Holbrook & Schumm, 1999; Schumm, 1977). It should be noted that variability in these factors was not included in our model, although they may influence the way a meandering river responds to faulting.

Finally, our model does not include all processes that influence the response of a river to a faulting perturbation. For instance, co‐seismic displacement may lead to bank failure or sand wells in the river channel (Fuller, 1912; Schumm et al., 2002). These perturbations influence morphodynamics and morphology by the local increase of sediment input to the river channel, or in the form of a temporary blockage of the flow. It was, for instance, shown for the Baghmati River in India that faulting leads to the formation of compressed meanders upstream of the fault (Jain & Sinha, 2005). These compressed meanders were subsequently abandoned as a result of channel straightening due to hydrological adjustments of the river channel (Jain & Sinha, 2005). Moreover, vertical displacement along a fault can lead to juxtaposition of different lithologies, causing variability in erosion resistance of the riverbed and banks over a fault (zone). Jin and Schumm (1987) showed, based on an experiment with a resistant clay block in the middle of the flume, that the presence of lithological differences leads to compression of meander bends, in this case upstream of the clay plug. Although differential erodibility due to juxtaposition was not included in our model setup, it can provide valuable opportunities for future modelling studies.

### Tectonic deformation rates

The faulting offsets that were used for the modelling scenarios are relatively large. The vertical displacements of 1/8th and 3/8th channel depth of the model correspond to offsets of 0.4 and 1.2 m for the model river. These offsets relate to a moment magnitude of ~6.5–6.8 (Wells & Coppersmith, 1994). These vertical displacements and earthquake magnitudes form, for example, the upper limit of the earthquakes in the LRE (Camelbeeck et al., 2007), which is the area on which our modelling scenarios are inspired. The displacement rates of 4 and 12 mm/(flood) year for the gradual displacement scenarios are very high compared to natural deformation rates at intraplate fault systems. Long‐term vertical displacement rates in the LRE did, for example, not exceed 0.1 mm/year (Vanneste et al., 2013). Nevertheless, the offset rates in this model study are within the range of summarized worldwide rates of Quaternary vertical deformation, which lie between 0.1 and 10 mm/year (Kaizuka, 1967; Ouchi, 1985; Schumm et al., 2000). Therefore, the gradual offset rates can be regarded as a reasonable upper estimation for active aseismic deformation to study the effects on river morphodynamics.

Our model results suggest that a vertical displacement of 1/8th of the river channel depth is sufficient to cause a morphodynamic response that is traceable in the geomorphological record compared to a reach that is not affected by faulting (Table 3). Similar relative offsets of river channel depth in an analogue experiment by Ouchi (1985) resulted in a response of the meandering channel as well. Their results show that an increase in slope results in an increased sinuosity of the experimental river compared to the control. However, scaling issues may complicate direct comparison between model results and natural river responses concerning deformation rates and the amount of vertical displacement. For example, observations of geomorphological records show that natural river systems already respond to deformation rates of ~1 mm/year (Zámolyi et al., 2010). Hence, although our model runs (faulting vs. control) give a solid indication of the morphodynamic responses of a river with downstream migrating meanders to faulting, direct comparison to natural rivers and deformation rates should be regarded carefully.

### Relative timescales

**FIGURE 17:**
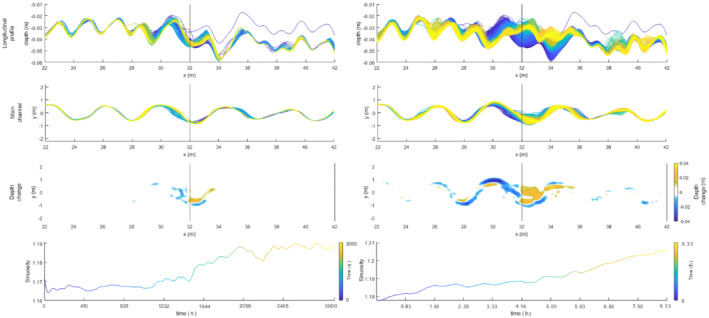
Evolution of the longitudinal bed profile, main channel position and sinuosity during the first 50 min (3000 s, left panels) and after 8.3 h (right panels) subsequent to a subsidence faulting perturbation [Colour figure can be viewed at wileyonlinelibrary.com]

Our model results show that the vertical adaption of the riverbed occurs relatively fast (within 4.17 h after the displacement; Figure 17) compared to the lateral adaption process (between 16.7 and 75 h). This discrepancy is best explained by the relations between the characteristic spatial scale and formative timescale of the morphological phenomena (Kleinhans et al., 2015), where in‐channel morphodynamics are an order of magnitude faster than bend migration and planform changes. For example, Ten Brinke (2004) showed that erosion of the IJssel River (NL) bed of ~0.5 to 1.5 m occurred within a few years after channel canalization that cut off several meander bends.

For the subsidence scenarios, it was shown that lateral channel migration rates increased by 1.5 to 23 times compared to the reference scenario without faulting (Table 3). This increase in lateral dynamics is the result of an enhanced fluvial gradient, caused by the fault offset, which causes higher flow‐velocity conditions, increased bank erosion and, hence, more lateral displacement and a sinuosity increase (Figure 16). Relatively reduced vertical and lateral displacement rates are linked to the uplift scenarios (Table 3), where fluvial gradient is reduced due to a developing backwater in front of the displaced fault (Figure 16).

Thus, our study shows that faulting increases or decreases both the vertical and lateral dynamics of a river channel by an order of magnitude compared to a non‐affected river reach. However, comparison of modeling results to natural river systems should be done with caution because of uncertainties related to temporal scaling. Moreover, the interaction between bar formation, bank erosion, overbank deposition and (riparian) vegetation is crucial in the morphodynamics of meandering rivers. Hence, different model settings may have affected the outcome of our model runs significantly, which should be taken into account when comparing our model results to natural river systems. This does not affect, however, conclusions derived from comparisons between the model runs.

### Transient response, fault location and preservation potential

Our model results, compared with the controls, show that a fault displacement of an alluvial meandering river with downstream migrating meander bends results in multiple, time‐dependent, morphological adjustments of the river channel such as erosion and sedimentation along the longitudinal river profile, sinuosity increase and (chute) cutoff of the channel (Figures 4, 7, 14, and 16). Therefore, the response is transient. This time‐dependent river response is important when deriving relationships between faulting and river dynamics from morphological and sedimentological records. It is crucial to understand if an observed morphological response is the final outcome of the morphodynamic adaption or part of an ongoing transient response. For example, sediment aggradation at the hanging wall normally leads to a locally increased preservation potential. However, only the lower parts of the sedimentary succession will be preserved if aggradation occurs alongside lateral migration (Lewin & Macklin, 2003; Toonen et al., 2012). This process can hence lead to a burial and masking of the geomorphology of the initial part of the sequence of responses to faulting. Alternatively, if a chute cutoff leads to incision of the new chute channel, the initial sinuosity increase may be partially preserved in the sedimentological and morphological record (Figure 16). However, the preservation of geomorphological and sedimentological responses to faulting also depends on the difference in relative timescales of river dynamics and faulting. A highly dynamic river is more likely to rework potential geomorphic indicators between successive faulting events than a river that is less dynamic. Moreover, the relative position at which a meander bend crosses a fault influences the river response to faulting (Figures 14, 15, 16). This is important to consider when analysing the geomorphological record for possible fault presence and evidence of faulting, as the timing of faulting is critical for the morphodynamic response of a downstream migrating meandering river.

The above indicates that without a detailed reconstruction of the response of a meandering channel, our abductive inferences from the morphological or sedimentological records might lead to erroneous conceptual models of the relation between faulting and alluvial river response. Our model results provide guideline to include process‐based reasoning in the interpretation of geomorphological and sedimentological observations of fluvial response to faulting. The combination of these approaches will help to understand the natural processes involved in more detail and might therefore lead to better predictions of possible effects of faulting on river morphodynamics.

## CONCLUSIONS

The numerical modelling of the morphodynamic response of an alluvial meandering river to various faulting scenarios in this study showed longitudinal and lateral adaptions of the channel. After an initial longitudinal riverbed adaption, sinuosity of the river channel increases as a result of the faulting‐enhanced valley gradient at the fault, which causes enhanced bank erosion and lateral displacement. This upward causation from the bend scale to the reach and floodplain scale arises from the complex interactions between meandering and floodplain and the nonlinearities of the sediment transport and chute cutoff processes.

Both the longitudinal and lateral adaptions are restricted to a zone of approximately 2 meander wavelengths around the fault for the applied offsets and diminish both upstream and downstream of the fault. The effects of fault‐bounded vertical deformation on meandering rivers are, hence, relatively local around the fault zone.

The transient response to faulting should be considered in the interpretation of morphological and sedimentological observations as it is crucial to understand if an observed morphological response is the final outcome of the morphodynamic adaption or part of an ongoing transient response.

Channel sinuosity and the pacing of morphological responses are highly sensitive to the relative position within a meander bend at which faulting occurs. Fault locations that enhance flow velocities over the point bar result in a faster sinuosity increase and subsequent chute cutoff than locations that enhance flow velocity towards the floodplain. As a result, in a meandering river system with downstream migrating bends, the timing of faulting is decisive for the morphodynamic response of the river to faulting.

## Data Availability

Model results output files are available on request from the corresponding author.

## References

[esp5315-bib-0001] Alexander, J. & Leeder, M.R. (1987) Active tectonic control on alluvial architecture. In: Ethridge, F.G. , Flores, R.M. & Harvey, M.D. (Eds.) Recent Developments in Fluvial Sedimentology. Tulsa, OK: Society for Sedimentary Geology, pp. 243–244. Special Publications of the SEPM, Vol. 39

[esp5315-bib-0002] Allen, J.R.L. (1978) Studies in fluviatile sedimentation: An exploratory quantitative model for the architecture of avulsion‐controlled alluvial suites. Sedimentary Geology, 21(2), 129–147. Available from: 10.1016/0037-0738(78)90002-7

[esp5315-bib-0003] Bridge, J.S. & Leeder, M.R. (1979) A simulation model of alluvial stratigraphy. Sedimentology, 26(5), 617–644. Available from: 10.1111/j.1365-3091.1979.tb00935.x

[esp5315-bib-0004] Bryant, M. , Falk, P. & Paola, C. (1995) Experimental study of avulsion frequency and rate of deposition. Geology, 23(4), 365–368. Available from: 10.1130/0091-7613(1995)023<0365:ESOAFA>2.3.CO;2

[esp5315-bib-0005] Camelbeeck, T. , Vanneste, K. , Alexandre, P. , Verbeeck, K. , Petermans, T. , Rosset, P. & Mazzotti, S. (2007) Relevance of active faulting and seismicity studies to assessments of long‐term earthquake activity and maximum magnitude in intraplate northwest Europe, between the Lower Rhine Embayment and the North Sea. In: Stein, S. & Mazzotti, S. (Eds.) Continental Intraplate Earthquakes: Science, Hazard, and Policy Issues. Boulder, CO: Geological Society of America, pp. 193–224. Special Paper No. 425

[esp5315-bib-0006] Crosato, A. (2007). Variations of channel migration rates in two meander models. In Proceedings of the 5th IAHR Symposium on River, Coastal and Estuarine Morphodynamics, Enschede, the Netherlands.

[esp5315-bib-0007] Crosato, A. (2009) Physical explanations of variations in river meander migration rates from model comparison. Earth Surface Processes and Landforms, 34(15), 2078–2086. Available from: 10.1002/esp.1898

[esp5315-bib-0008] Friedkin, J.F. (1945) A Laboratory Study of the Meandering of Alluvial Rivers. Vicksburg, MS: US Waterways Experiment Station.

[esp5315-bib-0009] Fuller, M.L. (1912) The New Madrid Earthquake. U.S. Geological Survey Bulletin 494. Reston, VA: U.S. Geological Survey.

[esp5315-bib-0010] Gasparini, N.M. , Fischer, G.C. , Adams, J.M. , Dawers, N.H. & Janoff, A.M. (2016) Morphological signatures of normal faulting in low‐gradient alluvial rivers in south‐eastern Louisiana, USA. Earth Surface Processes and Landforms, 41(5), 642–657. Available from: 10.1002/esp.3852

[esp5315-bib-0011] Gomez, B. & Marron, D.C. (1991) Neotectonic effects on sinuosity and channel migration, Belle Fourche River, western South Dakota. Earth Surface Processes and Landforms, 16(3), 227–235. Available from: 10.1002/esp.3290160304

[esp5315-bib-0013] Hickin, E.J. & Nanson, G. (1975) The character of channel migration on the Beatton River, northeast British Columbia, Canada. Bulletin of the Geological Society of America, 86(4), 487–494. Available from: 10.1130/0016-7606(1975)86<487:TCOCMO>2.0.CO;2

[esp5315-bib-0014] Holbrook, J. & Schumm, S.A. (1999) Geomorphic and sedimentary response of rivers to tectonic deformation: A brief review and critique of a tool for recognizing subtle epeirogenic deformation in modern and ancient settings. Tectonophysics, 305(1–3), 287–306. Available from: 10.1016/S0040-1951(99)00011-6

[esp5315-bib-0015] Hooke, J.M. (2007a) Spatial variability, mechanisms and propagation of change in an active meandering river. Geomorphology, 84(3–4), 277–296. Available from: 10.1016/j.geomorph.2006.06.005

[esp5315-bib-0016] Hooke, J.M. (2007b) Complexity, self‐organisation and variation in behaviour in meandering rivers. Geomorphology, 91(3–4), 236–258. Available from: 10.1016/j.geomorph.2007.04.021

[esp5315-bib-0017] Houtgast, R.F. , van Balen, R.T. & Kasse, C. (2005) Late Quaternary evolution of the Feldbiss Fault (Roer Valley Rift System, the Netherlands) based on trenching, and its potential relation to glacial unloading. Quaternary Science Reviews, 24(3–4), 489–508. Available from: 10.1016/j.quascirev.2004.01.012

[esp5315-bib-0018] Howard, A.D. (1992) Modelling channel migration and floodplain sedimentation in meandering streams. In: Carling, P.A. & Petts, G.E. (Eds.) Lowland Floodplain Rivers. Chichester: Wiley, pp. 1–41.

[esp5315-bib-0019] Howard, A.D. & Knutson, T.R. (1984) Sufficient conditions for river meandering: A simulation approach. Water Resources Research, 20(11), 1659–1667. Available from: 10.1029/WR020i011p01659

[esp5315-bib-0020] Iwasaki, T. , Shimizu, Y. & Kimura, I. (2016) Numerical simulation of bar and bank erosion in a vegetated floodplain: A case study in the Otofuke River. Advances in Water Resources, 93, 118–134. Available from: 10.1016/j.advwatres.2015.02.001

[esp5315-bib-0021] Jain, V. & Sinha, R. (2005) Response of active tectonics on the alluvial Baghmati River, Himalayan foreland basin, eastern India. Geomorphology, 70(3–4), 339–356. Available from: 10.1016/j.geomorph.2005.02.012

[esp5315-bib-0022] Jin, D. & Schumm, S.A. (1987) A new technique for modeling river morphology. In: Gardner, V. (Ed.) International Geomorphology. Chichester: Wiley, pp. 681–690.

[esp5315-bib-0023] Jorgensen, D.W . (1990) *Adjustment of alluvial river morphology and process to localized active tectonics*. PhD thesis, Colorado State University, USA.

[esp5315-bib-0067] Kaizuka, S. (1967) Rate of folding in the Quaternary and the present. *Geographical Reports of Tokyo Metropolitan University*, 2, 1–10.

[esp5315-bib-0024] Kemna, H.A . (2005) *Pliocene and Lower Pleistocene Stratigraphy in the Lower Rhine Embayment, Germany*. PhD thesis, Universität Köln, Germany.

[esp5315-bib-0025] Kleinhans, M.G. (2010) Sorting out river channel patterns. Progress in Physical Geography, 34(3), 287–326. Available from: 10.1177/0309133310365300

[esp5315-bib-0026] Kleinhans, M.G. , Braudrick, C. , van Dijk, W.M. , van de Lageweg, W.I. , Teske, R. & van Oorschot, M. (2015) Swiftness of biomorphodynamics in Lilliput‐to‐giant‐sized rivers and deltas. Geomorphology, 244, 56–73. Available from: 10.1016/j.geomorph.2015.04.022

[esp5315-bib-0027] Kleinhans, M.G. , Ferguson, R.I. , Lane, S.N. & Hardy, R.J. (2013) Splitting rivers at their seams: Bifurcations and avulsion. Earth Surface Processes and Landforms, 38(1), 47–61. Available from: 10.1002/esp.3268

[esp5315-bib-0068] Kleinhans, M.G. , Van Dijk, W.M. , Van de Lageweg, W.I. , Hoyal, D.C.J.D. , Markies, H. , Van Maarseveen, M. et al. (2014) Quantifiable effectiveness of experimental scaling of river‐ and delta morphody‐namics and stratigraphy. Earth‐Science Reviews, 133, 43–61.

[esp5315-bib-0029] Lancaster, S.T. & Bras, R.L. (2002) A simple model of river meandering and its comparison to natural channels. Hydrological Processes, 16(1), 1–26. Available from: 10.1002/hyp.273

[esp5315-bib-0030] Lanzoni, S. & Seminara, G. (2006) On the nature of meander instability. Journal of Geophysical Research: Earth Surface, 111(F4), 1–14. 10.1029/2005JF000416 20411040

[esp5315-bib-0031] Leopold, L.B. , Wolman, M.G . (1957) *River channel patterns: braided, meandering, and straight*. U.S. Government Printing Office: Washington, DC.

[esp5315-bib-0032] Leopold, L.B. & Wolman, M.G. (1960) River meanders. Geological Society of America Bulletin, 71(6), 769–793. Available from: 10.1130/0016-7606(1960)71[769:RM]2.0.CO;2

[esp5315-bib-0033] Lewin, J. & Macklin, M.G. (2003) Preservation potential for Late Quaternary river alluvium. Journal of Quaternary Science, 18(2), 107–120. Available from: 10.1002/jqs.738

[esp5315-bib-0034] Mackey, S.D. & Bridge, J.S. (1995) Three‐dimensional model of alluvial stratigraphy: Theory and application. Journal of Sedimentary Research, B65(1b), 7–31. Available from: 10.1306/D42681D5-2B26-11D7-8648000102C1865D

[esp5315-bib-0035] Meyer‐Peter, E. & Müller, R. (1948) Formulas for bed‐load transport. In: Proceedings of the 2nd IAHSR Meeting. Beijing: IAHR, pp. 39–64.

[esp5315-bib-0036] Mosselman, E. (1995) A review of mathematical models of river planform changes. Earth Surface Processes and Landforms, 20(7), 661–670. Available from: 10.1002/esp.3290200708

[esp5315-bib-0037] Ouchi, S. (1985) Response of alluvial rivers to slow active tectonic movement. Geological Society of America Bulletin, 96(4), 504–515. Available from: 10.1130/0016-7606(1985)96<504:ROARTS>2.0.CO;2

[esp5315-bib-0038] Parker, G . (2004) 1D sediment transport morphodynamics with applications to rivers and turbidity currents . https://csdms.colorado.edu/wiki/1D_Sediment_Transport_Morphodynamics_with_applications_to_Rivers_and_Turbidity_Currents

[esp5315-bib-0039] Parker, G. , Shimizu, Y. , Wilkerson, G.V. , Eke, E.C. , Abad, J.D. , Lauer, J.W. , Paola, C. , Dietrich, W.E. & Voller, V.R. (2011) A new framework for modeling the migration of meandering rivers. Earth Surface Processes and Landforms, 36(1), 70–86. Available from: 10.1002/esp.2113

[esp5315-bib-0040] Petrovszki, J. , Székely, B. & Timár, G. (2012) A systematic overview of the coincidences of river sinuosity changes and tectonically active structures in the Pannonian Basin. Global and Planetary Change, 98, 109–121. Available from: 10.1016/j.gloplacha.2012.08.005

[esp5315-bib-0041] Schumm, S.A. (1973) Geomorphic thresholds and complex response of drainage systems. Fluvial Geomorphology, 6, 69–85.

[esp5315-bib-0042] Schumm, S.A. (1977) The Fluvial System. New York: Wiley.

[esp5315-bib-0043] Schumm, S.A. , Dumont, J.F. & Holbrook, J.M. (2002) Active Tectonics and Alluvial Rivers. Cambridge: Cambridge University Press.

[esp5315-bib-1043] Schumm, S.A. , Dumont, J.F. & Holbrook, J.M. (2000) Active Tectonics and Alluvial Rivers (Vol. 276). Cambridge: Cambridge University Press.

[esp5315-bib-0044] Schumm, S.A. & Khan, H.R. (1972) Experimental study of channel patterns. Geological Society of America Bulletin, 83(6), 1755–1770. Available from: 10.1130/0016-7606(1972)83[1755:ESOCP]2.0.CO;2

[esp5315-bib-0045] Seminara, G. (2006) Meanders. Journal of Fluid Mechanics, 554(1), 271–297. Available from: 10.1017/S0022112006008925

[esp5315-bib-0046] Shimizu, Y. , Inoue, T. , Hamaki, M. & Iwasaki, T. (2013) iRIC Software, Changing River Science: Nays2D Solver Manual. Sapporo: International River Interface Cooperative.

[esp5315-bib-0047] Stølum, H.H. (1996) River meandering as a self‐organization process. Science, 271(5256), 1710–1713. Available from: 10.1126/science.271.5256.1710

[esp5315-bib-0048] Sun, T. , Meakin, P. , Jøssang, T. & Schwarz, K. (1996) A simulation model for meandering rivers. Water Resources Research, 32(9), 2937–2954. Available from: 10.1029/96WR00998

[esp5315-bib-0049] Sylvester, Z. , Durkin, P. & Covault, J.A. (2019) High curvatures drive river meandering. Geology, 47(3), 263–266. Available from: 10.1130/G45608.1

[esp5315-bib-0050] Ten Brinke, W. (2004) The Dutch Rhine, a Restrained River. Diemen: Veen Magazines B.V.

[esp5315-bib-0051] Timár, G. (2003) Controls on channel sinuosity changes: A case study of the Tisza River, the Great Hungarian Plain. Quaternary Science Reviews, 22(20), 2199–2207. Available from: 10.1016/S0277-3791(03)00145-8

[esp5315-bib-0052] Toonen, W.H. , Kleinhans, M.G. & Cohen, K.M. (2012) Sedimentary architecture of abandoned channel fills. Earth Surface Processes and Landforms, 37(4), 459–472. Available from: 10.1002/esp.3189

[esp5315-bib-0053] van Balen, R.T. , Bakker, M.A.J. , Kasse, C. , Wallinga, J. & Woolderink, H.A.G. (2019) A Late Glacial surface rupturing earthquake at the Peel Boundary fault zone, Roer Valley Rift System, the Netherlands. Quaternary Science Reviews, 218, 254–266. Available from: 10.1016/j.quascirev.2019.06.033

[esp5315-bib-0054] van Balen, R.T. , Houtgast, R.F. & Cloetingh, S.A.P.L. (2005) Neotectonics of the Netherlands: A review. Quaternary Science Reviews, 24(3–4), 439–454. Available from: 10.1016/j.quascirev.2004.01.011

[esp5315-bib-0057] van Dijk, W.M. , van de Lageweg, W.I. & Kleinhans, M.G. (2012) Experimental meandering river with chute cutoffs. Journal of Geophysical Research: Earth Surface, 117(F3), 1–18. 10.1029/2011JF002314

[esp5315-bib-0058] Vanneste, K. , Camelbeeck, T. & Verbeeck, K. (2013) A model of composite seismic sources for the Lower Rhine Graben, Northwest Europe. Bulletin of the Seismological Society of America, 103(2A), 984–1007. Available from: 10.1785/0120120037

[esp5315-bib-0059] Weiss, S.F . (2016) *Meandering river dynamics* PhD thesis, University of Illinois at Urbana‐Champaign, USA.

[esp5315-bib-0060] Weisscher, S.A. , Shimizu, Y. & Kleinhans, M.G. (2019) Upstream perturbation and floodplain formation effects on chute‐cutoff‐dominated meandering river pattern and dynamics. Earth Surface Processes and Landforms, 44(11), 2156–2169. Available from: 10.1002/esp.4638 31598027 PMC6774324

[esp5315-bib-0061] Wells, D.L. & Coppersmith, K.J. (1994) New empirical relationships among magnitude, rupture length, rupture width, rupture area, and surface displacement. Bulletin of the Seismological Society of America, 84(4), 974–1002.

[esp5315-bib-0062] Westerhoff, W.E. , Kemna, H.A. & Boenigk, W. (2008) The confluence area of Rhine, Meuse, and Belgian rivers: Late Pliocene and Early Pleistocene fluvial history of the northern Lower Rhine Embayment. Netherlands Journal of Geosciences‐Geologie en Mijnbouw, 87(1), 107–125. Available from: 10.1017/S0016774600024070

[esp5315-bib-0063] Woolderink, H.A.G. , Cohen, M.K. , Kleinhans, M.G. & van Balen, R.T. (2021) Patterns in river channel sinuosity of the Meuse, Roer and Rhine rivers in the Lower Rhine Embayment rift system, are they tectonically forced? Geomorphology, 375, 1–12. 10.1016/j.geomorph.2020.107550

[esp5315-bib-0064] Woolderink, H.A.G. , Kasse, C. , Cohen, K.M. , Hoek, W.Z. & van Balen, R.T. (2018) Spatial and temporal variations in river terrace formation, preservation, and morphology in the Lower Meuse Valley, the Netherlands. Quaternary Research, 91(2), 548–569. Available from: 10.1017/qua.2018.49

[esp5315-bib-0065] Zámolyi, A. , Székely, B. , Draganits, E. & Timár, G. (2010) Neotectonic control on river sinuosity at the western margin of the Little Hungarian Plain. Geomorphology, 122(3–4), 231–243. Available from: 10.1016/j.geomorph.2009.06.028

